# Snapshots of pre-rRNA structural flexibility reveal eukaryotic 40S assembly dynamics at nucleotide resolution

**DOI:** 10.1093/nar/gku815

**Published:** 2014-09-08

**Authors:** Ralph D. Hector, Elena Burlacu, Stuart Aitken, Thierry Le Bihan, Maarten Tuijtel, Alina Zaplatina, Atlanta G. Cook, Sander Granneman

**Affiliations:** 1 Centre for Synthetic and Systems Biology (SynthSys), University of Edinburgh, Edinburgh, EH9 3JD, UK; 2 Wellcome Trust Centre for Cell Biology, University of Edinburgh, Edinburgh, EH9 3JR, UK; 3 MRC Human Genetics Unit, University of Edinburgh, Western General Hospital, Crewe Road, Edinburgh, EH4 2XU, UK

## Abstract

Ribosome assembly in eukaryotes involves the activity of hundreds of assembly factors that direct the hierarchical assembly of ribosomal proteins and numerous ribosomal RNA folding steps. However, detailed insights into the function of assembly factors and ribosomal RNA folding events are lacking. To address this, we have developed ChemModSeq, a method that combines structure probing, high-throughput sequencing and statistical modeling, to quantitatively measure RNA structural rearrangements during the assembly of macromolecular complexes. By applying ChemModSeq to purified 40S assembly intermediates we obtained nucleotide-resolution maps of ribosomal RNA flexibility revealing structurally distinct assembly intermediates and mechanistic insights into assembly dynamics not readily observed in cryo-electron microscopy reconstructions. We show that RNA restructuring events coincide with the release of assembly factors and predict that completion of the head domain is required before the Rio1 kinase enters the assembly pathway. Collectively, our results suggest that 40S assembly factors regulate the timely incorporation of ribosomal proteins by delaying specific folding steps in the 3′ major domain of the 20S pre-ribosomal RNA.

## INTRODUCTION

Ribosome synthesis in eukaryotes is an incredibly complex process that requires around 200 ribosome assembly factors to facilitate the modification, folding and processing of rRNA precursors (pre-rRNA) and their ordered assembly with r-proteins (1). In the yeast *Saccharomyces cerevisiae*, ribosome assembly starts in the nucleolus, where a 35S rRNA precursor is cleaved at sites A_0_, A_1_ and A_2_ within a large, 90S-sized complex. This results in the formation of a pre-60S particle that contains the 27SA_2_ pre-rRNA and a pre-40S particle that contains a 3′ extended 18S molecule called the 20S pre-rRNA. The pre-40S complexes are rapidly exported to the cytoplasm where cleavage at site D generates the mature 18S rRNA. During maturation, several nucleotides in the 20S pre-rRNA are enzymatically modified. Shortly after export to the cytoplasm, Dim1 methylates two adenosines near the D-site (2). Just before D-site cleavage, an aminocarboxypropyl (acp) group is added to a hypermodified uridine at position 1191 in the P-site of the 18S rRNA decoding center (3). This hypermodification is important for efficient translation and D-site cleavage (4).

**Figure 1.**
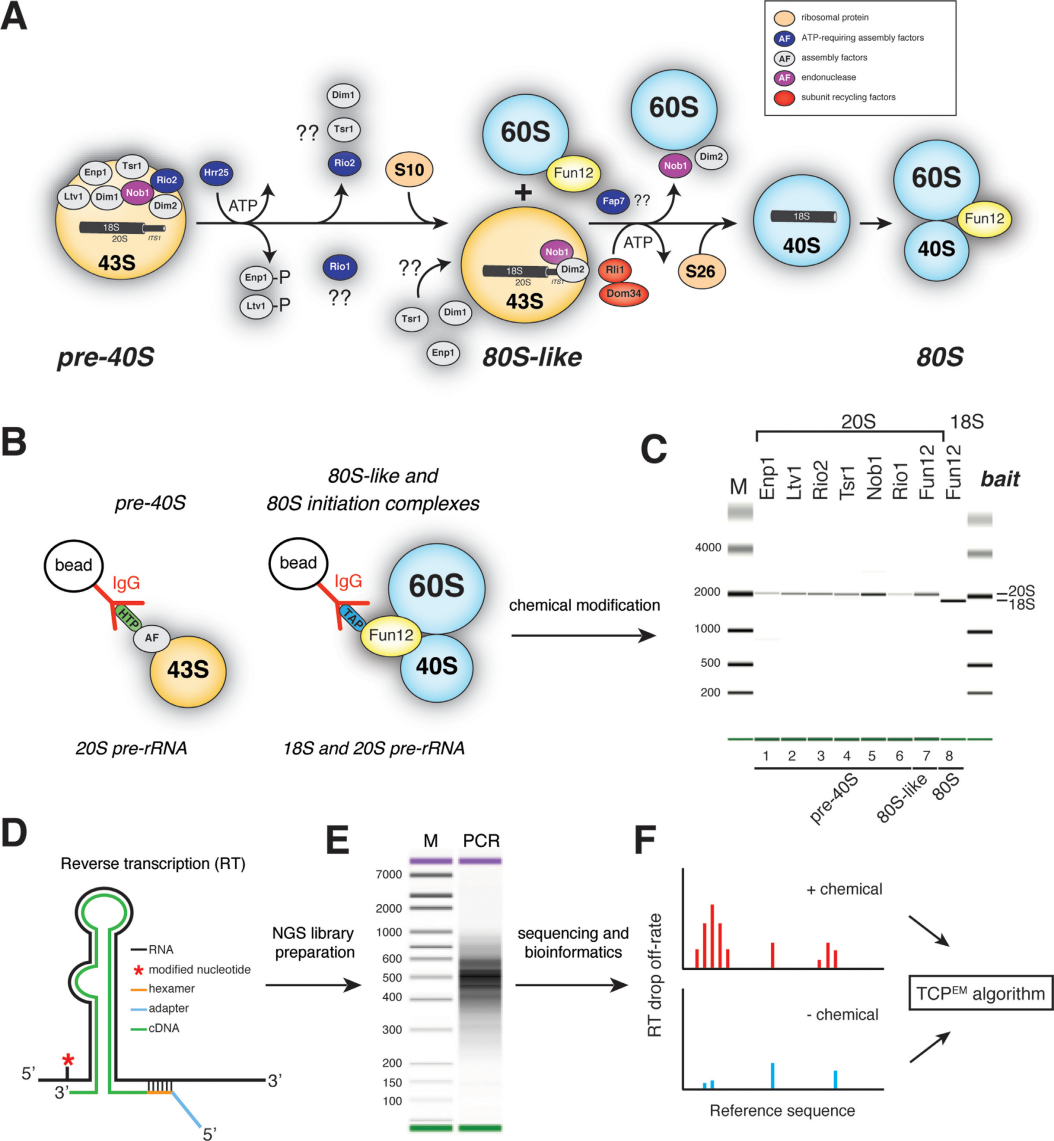
40S subunit maturation in *Saccharomyces cerevisiae*. (**A**) Schematic representation of the cytoplasmic 40S synthesis pathway. (**B** and **C**) Pre-40S particles at different stages of assembly are purified using strains expressing TAP-or HTP-tagged assembly factors. Particles are chemically modified (B), RNA is extracted and gel purified 20S and 18S rRNAs are analyzed on a Bioanalyzer (C). (**D** and **E**) cDNA libraries, generated by random priming, were PCR amplified and (**E**) sequenced on a HiSeq. (**F**) Chemically modified samples were compared to solvent treated samples using a two-channel Poisson expectation maximization algorithm (TCMP^EM^) to identify nucleotides that had the highest likelihood of being modified.

Figure 1A shows a schematic representation of our current knowledge about the cytoplasmic steps of yeast 40S maturation. As in bacteria, small subunit assembly in yeast involves many restructuring and/or remodeling steps; however, these events are still poorly understood. The earliest pre-40S complexes contain an almost complete set of r-proteins, a handful of assembly factors and the endonuclease Nob1 that performs the final cleavage event at site D (Figure 1A). The majority of assembly factors associate with pre-40S complexes in the nucleus and, upon entry into the cytoplasm, are believed to progressively dissociate from intermediates. However, it is not entirely clear in which order these factors are released (Figure 1A). CRAC UV cross-linking and cryo-electron microscopy (cryo-EM) experiments have localized binding sites for many assembly factors (5,6). Several assembly factors cross-linked near r-protein binding sites and it was proposed that this could interfere with stable binding of r-proteins to pre-40S (5). Their presence is also incompatible with binding of mRNA, tRNAs and translation initiation factors, indicating that they block premature translation initiation (5,6).

Hrr25 phosphorylation of Ltv1 and Enp1 was shown to trigger a cascade of events that results in their release from pre-40S complexes (7), and this is likely the first cytoplasmic remodeling step (8) (Figure 1A). Whether Rio1 interacts with pre-40S complexes in the nucleus or the cytoplasm is still under debate. Although Rio1 is predominantly cytoplasmic, it shuttles between the nucleus and the cytoplasm (9). Translation initiation factor eIF5B/Fun12 stimulates processing at site D by promoting the joining of a pre-40S particle to a 60S subunit, forming an 80S-like complex (10–12) (Figure 1A). In the current model this step ensures that Nob1 only cleaves in pre-40S particles that are able to adopt a translation-competent conformation. Precisely when the other assembly factors dissociate is not fully understood. Yeast Enp1, Tsr1, Nob1, Dim1 and Dim2 co-sedimented with stalled 80S-like complexes in density gradients (10), suggesting that these proteins remain associated with pre-40S complexes until the very final stages. In contrast, work in human cells indicated that only hNob1 and hDim2 are present in late particles (13).

Despite tremendous progress over the years, there is still much to be learned about the function of ribosome assembly factors, in particular how they influence rRNA folding steps. Using cryo-electron microscopy, the Böttcher and Hurt laboratories demonstrated a role for Hrr25 in the formation of the 18S rRNA ‘beak’ structure, which is formed by a protruding helix in the 18S rRNA (7). CRAC UV cross-linking experiments have also indicated that ribosome assembly factors could contribute to the formation of the functional center of the ribosome (5). Unfortunately, high-resolution insights into rRNA folding events are lacking, making it difficult to obtain mechanistic insights into how assembly factors regulate this process. Atomic resolution structures of assembly intermediates would undoubtedly provide the most valuable insights into ribosome biogenesis, however, the instability and heterogeneity of purified ribosome assembly intermediates makes high-resolution structural studies extremely challenging.

As an alternative to direct structural approaches, we decided to establish protocols that would enable us to measure the flexibility of pre-rRNAs in ribosome assembly intermediates. This would provide high-resolution insights into rRNA restructuring events. SHAPE (Selective 2′-hydroxyl acylation analyzed by primer extension) is currently the gold standard method for mapping dynamic regions within RNA. SHAPE chemicals generically react with 2′-OH of all four nucleotides in single-stranded and/or flexible regions (14). Sites of 2′-O-adduct formation are identified as stops in reverse transcription (RT) reactions. SHAPE analysis of rRNA in crystallized bacterial ribosomes showed a strong correlation between nucleotide flexibility and SHAPE reactivity, independent of solvent or molecular accessibility (15). Because SHAPE provides a read-out of RNA dynamics (rather than solvent accessibility or protein association), it is ideally suited to measure RNA conformational changes during the assembly of macromolecular complexes, such as pre-ribosomes. We developed a high-throughput method called ChemModSeq that combines SHAPE with next-generation sequencing and statistical modeling. This quantitative approach allowed us to rapidly generate an overview of RNA restructuring events during 40S synthesis in yeast. Our results indicate that assembly factors regulate the timely assembly of ribosomal proteins by temporarily maintaining more flexible pre-rRNA conformations. We predict that this activity is required to prevent premature interactions that could lead to kinetic folding traps. We show that late restructuring in the 3′ major domain coincides with the release of assembly factors Ltv1, Enp1, Rio2 and Tsr1 in the cytoplasm. We propose that once this has been completed Rio1 enters the assembly pathway and 80S-like complexes can be formed.

Collectively, our results reveal new mechanistic insights into how ribosome assembly factors regulate the formation of 40S subunits in a eukaryote.

## MATERIALS AND METHODS

### Yeast strains and media

*Saccharomyces cerevisiae* strain BY4741 (MATa; *his3Δ1; leu2Δ0; met15Δ0; ura3Δ0*) was used as the parental strain (16). An overview of the strains used in this study is provided in Supplementary Table S3. The HTP (HIS6-TEV-2xProtA) and TAP carboxyl-tagged strains (Calmodulin binding peptide-TEV-2xProtA) were generated by polymerase chain reaction as described (17) (see Supplementary Table S4). Strains were grown in YPD (1% yeast extract, 2% peptone, 2% dextrose) or YPG/R (YP with 2% galactose and 2% raffinose) at 30°C. For the Nob1 and Fap7-depletion experiments, cells were grown in YPG/R to and OD_600_ of ∼0.5, shifted to YPD and grown for four hours to an OD_600_ of ∼1.0.

### Immunoprecipitation and chemical modification of rRNA

Immunoprecipitations performed with HTP-and TAP-tagged proteins were performed with TNM150 buffer (50 mM Tris–HCl pH 7.8, 1.5 mM MgCl_2_, 150 mM NaCl, 0.1% NP-40 and 5 mM β-mercaptoethanol), as previously described (18), using 1–5 g of cells as starting material. To immunoprecipitate FLAG-tagged r-proteins, TMN150 lysates prepared from 10 OD_600_ units of cells were incubated with 30 μl of anti-FLAG M2 magnetic beads (Sigma) for 1 h at 4°C. After washing the beads three times with 1 ml TMN150, RNA was extracted as described below. For *in vitro* modification reactions, pre-40S particles immobilized on 250 μl of IgG beads were resuspended in 200 μl of TMN150 buffer (without β-mercaptoethanol; final volume ∼400 μl). One-methyl-7-nitroisatoic anhydride (1M7; 15 mM final concentration) or DMSO (Dimethyl sulfoxide) (control; 5% final) was subsequently added to the beads and incubated for 3 min at room temperature. *In vitro* dimethyl sulfate (DMS) modification reactions were performed as previously described (15,19,20). For *in vivo* modification reactions with 2-methylnicotinic acid imidazolide (NAI), 1 l of cells was grown in YPD to an OD_600_ of ∼1.0. Cells were harvested, washed with phosphate-buffered saline (PBS) and resuspended in 4 ml of PBS per gram of cells. NAI was added to a final concentration of 100 mM and incubated for exactly 10 min at room temperature. Cells were washed with 50 ml of ice-cold PBS and pellets were snap frozen in liquid nitrogen. NAI was synthesized as previously described (21). RNA was extracted (see below). 20S and 18S pre-rRNAs were gel purified as previously described (19).

For analyses on *in vitro* refolded rRNA, gel purified 18S rRNA was refolded in TMN150 by heating the RNA at 65°C for 10 min and allowed to gradually cool to room temperature in a heating block. 1M7 was added to a final concentration of 15 mM (5% DMSO final) and incubated at room temperature for 3 min. RNA was extracted by phenol-chloroform extraction and precipitated by ethanol precipitation.

Free 40S subunits (devoid of mRNA, tRNA and initiation factors) were purified using a combination of published protocols (22–24). Briefly, cells were lysed in a high salt buffer (50 mM Tris pH 7.8, 500 mM KAc, 10 mM MgCl_2_, 0.2% Triton X-100 and 2 mM DTT (Dithiothreitol) ) and clarified extracts were incubated with GTP (Guanosine-5'-triphosphate) and puromycin (1 mM final each) for half an hour at 37°C. Extracts were then fractionated on 10–50% sucrose gradients. Fractions highly enriched for free 40S subunits were modified with 1M7 (15 mM final) or incubated with DMSO (5% final) for 5 min at room temperature. 18S RNA was extracted and analyzed by primer extension (for details, see Supplementary Data).

We designed the chemical probing conditions so that 1 in every 100–300 nucleotides was modified (25). We only selected RNA samples where, by primer extension, we could detect high levels of chemical modification but little signal decay over a stretch of ∼200 nucleotides. The quality and integrity of isolated rRNAs were analyzed on a 2100 Bionalyzer (Agilent) using an RNA Nano 6000 kit.

Additional methods can be found in the Supplementary Data online.

## RESULTS

### Purification and chemical modification of pre-40S intermediates

For the rRNA structure probing experiments, 40S assembly intermediates at various stages of maturation were affinity purified using yeast strains expressing HTP or TAP-tagged assembly factors (Figure 1A–C). Intriguingly, unlike the Rio1-TAP strain (26), the Rio1-HTP strain used for our studies did not show noticeable growth or pre-rRNA processing defects (Supplementary Figure S1A–C). TAP-tagged Fun12 was used as bait for 80S translation initiation complexes, a source of mature 40S subunits (Figure 1B). Using Fun12-TAP as bait, we also purified the recently described 80S-like complexes that accumulated in Fap7 depleted strains (10) (Supplementary Figure S1A, lane 8). The Nob1-TAP strain was included as it is severely delayed in D-site cleavage (5) (Supplementary Figure S1A and B) allowing us to determine whether stalled pre-40S complexes had any unusual structural features.

Bioanalyzer analyses showed that our purification protocol generated intact and highly pure pre-rRNA samples (Figure 1C).

### Development of ChemModSeq, an ultra-high-throughput RNA structure probing and analysis method

Recent technological advances have made it possible to obtain RNA secondary structure information by combining chemical or enzymatic probing with high-throughput sequencing (27–33). We developed a protocol, dubbed ChemModSeq that uses RT with oligonucleotides that randomly hybridize to the RNA template (Figure 1D), allowing us to quickly generate an overview of chemically modified sites in rRNA molecules in a single RT reaction. While this work was in progress, a similar method, called structure-seq, was described to measure mRNA structure transcriptome-wide in *Arabidopsis thaliana in vivo* (31). The main difference between our protocol and structure-seq is that we apply a probabilistic model to map sites of chemical modification with high accuracy. In addition, we paired-end sequenced the cDNAs to obtain RT drop-off positions and RT oligonucleotide annealing sites. For each nucleotide we then calculate RT drop-off rates, which we define as the total number of reads that stop at a nucleotide divided by the total number of reads covering that nucleotide. This provided an indication of how reactive to a chemical a particular nucleotide was. Data generated using the ChemModSeq and structure-seq protocols can be used to calculate chemical (DMS/SHAPE) reactivity values for each nucleotide (31). However this approach does not consider whether the observed drop-off counts in chemically modified RNA are statistically significantly over background. To address this we developed a two-channel Poisson expectation maximization (EM) algorithm (TCP^EM^; Figure 1F, see Supplementary Data) that calculates for each nucleotide the likelihood that it was chemically modified. In sequencing data from both unmodified and modified RNA there are nucleotide positions where the polymerase is more likely to drop-off (high), and positions where drop-off is less likely (low). The TCP^EM^ algorithm assumes that these high and low drop-off rates are approximately constant along the RNA and assigns each nucleotide position to one of three categories: high drop-off in both channels (stops not caused by chemical), low drop-off in both channels (unmodified), high drop-off in the modified channel and low in the control (modified). A Poisson model is then used to assess the likelihood of a drop-off count d_x_ for an inferred RNA-wide drop-off rate of λ. An EM algorithm then assigns each nucleotide position to one of the three categories.

To test the reliability of the ChemModSeq protocol we applied it to purified 80S translation initiation complexes, focusing on the 18S rRNA. This allowed us to compare the results with available crystal structures of yeast ribosomal subunits. To test the specificity of our approach, DMS was used as a chemical probe as it preferentially reacts with the N1 of adenosine and the N3 of cytosine. Biological replicates showed a high Pearson correlation for RT drop-off rates in the 18S rRNA (Supplementary Figure S2), demonstrating that the ChemModSeq protocol generates highly reproducible results.

**Figure 2.**
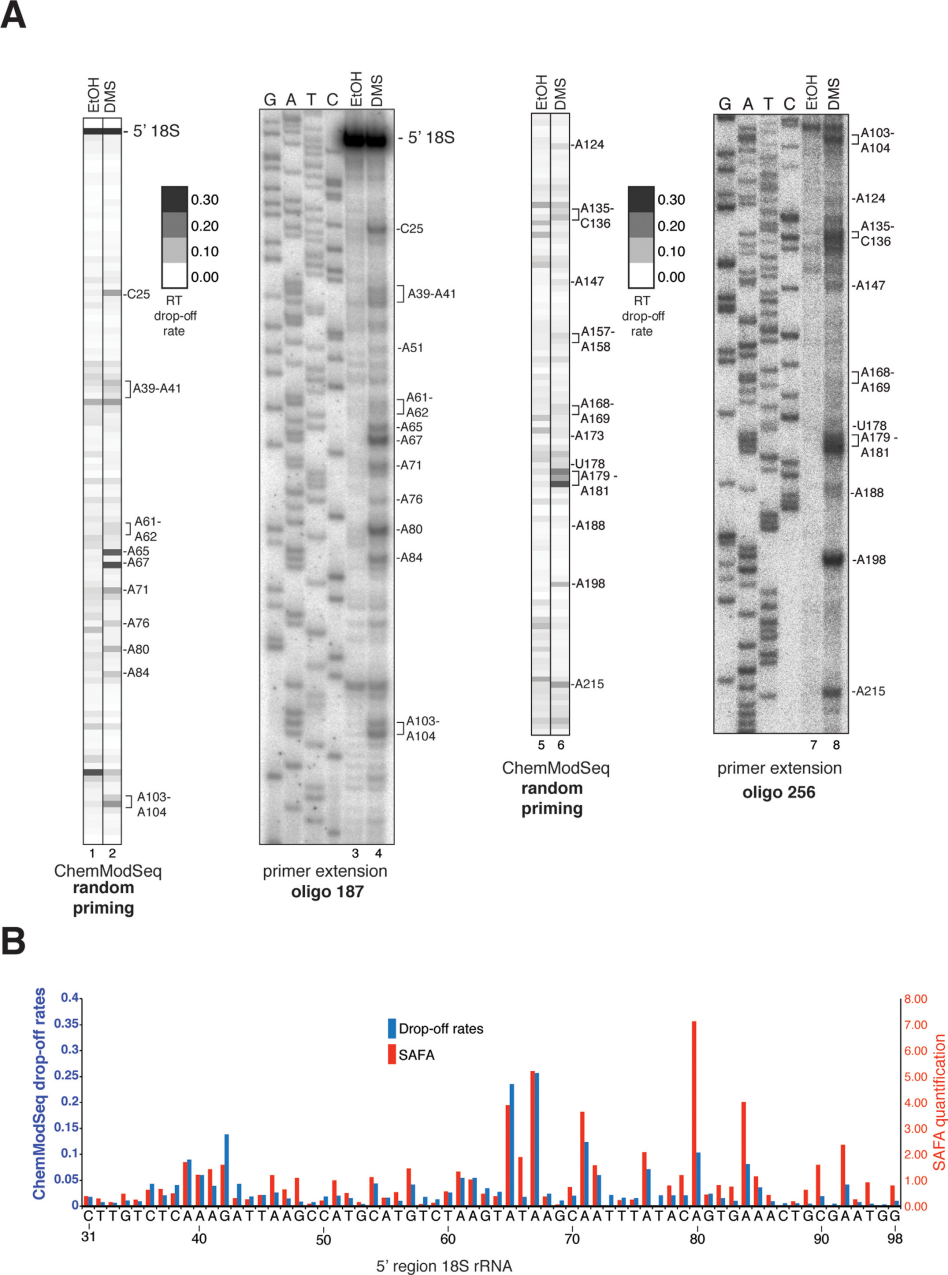
ChemModSeq accurately and quantitatively measures RNA secondary structure. (**A**) Primer extension results and ChemModSeq drop-off rates for DMS modified and unmodified 18S rRNA, focusing on the first ∼100 nucleotides. To compare ChemModSeq with primer extension data, drop-off rates were converted to gray scale heat maps in which each block represents a single nucleotide. The darker the color of the block, the higher the drop-off rate. Oligos used for primer extensions are indicated in bold (see Supplementary Table S4). The positions of the modified nucleotides are indicated on the right side of each panel. (**B**) ChemModSeq data agree well with primer extension results. Direct comparison of the primer extension results shown in (A), quantified using SAFA (34) (red bars) and ChemModSeq RT drop-off rates (blue bars) for the same region (nucleotides 31–98).

We next compared ChemModSeq RT drop-off rates to primer extension results quantified using the semi-automated footprinting analysis software (SAFA) (34), focusing on the 5′ end of the 18S rRNA (Figure 2). To allow direct visual comparison, RT drop-off rates were represented as grayscale heat maps (Figure 2A). This revealed that the two different approaches produced remarkably similar results; with statistical analysis confirming a strong correlation between the DMS reactivity values generated by SAFA and the ChemModSeq RT drop-off rates (Figure 2B; *r* = 0.77).

**Figure 3.**
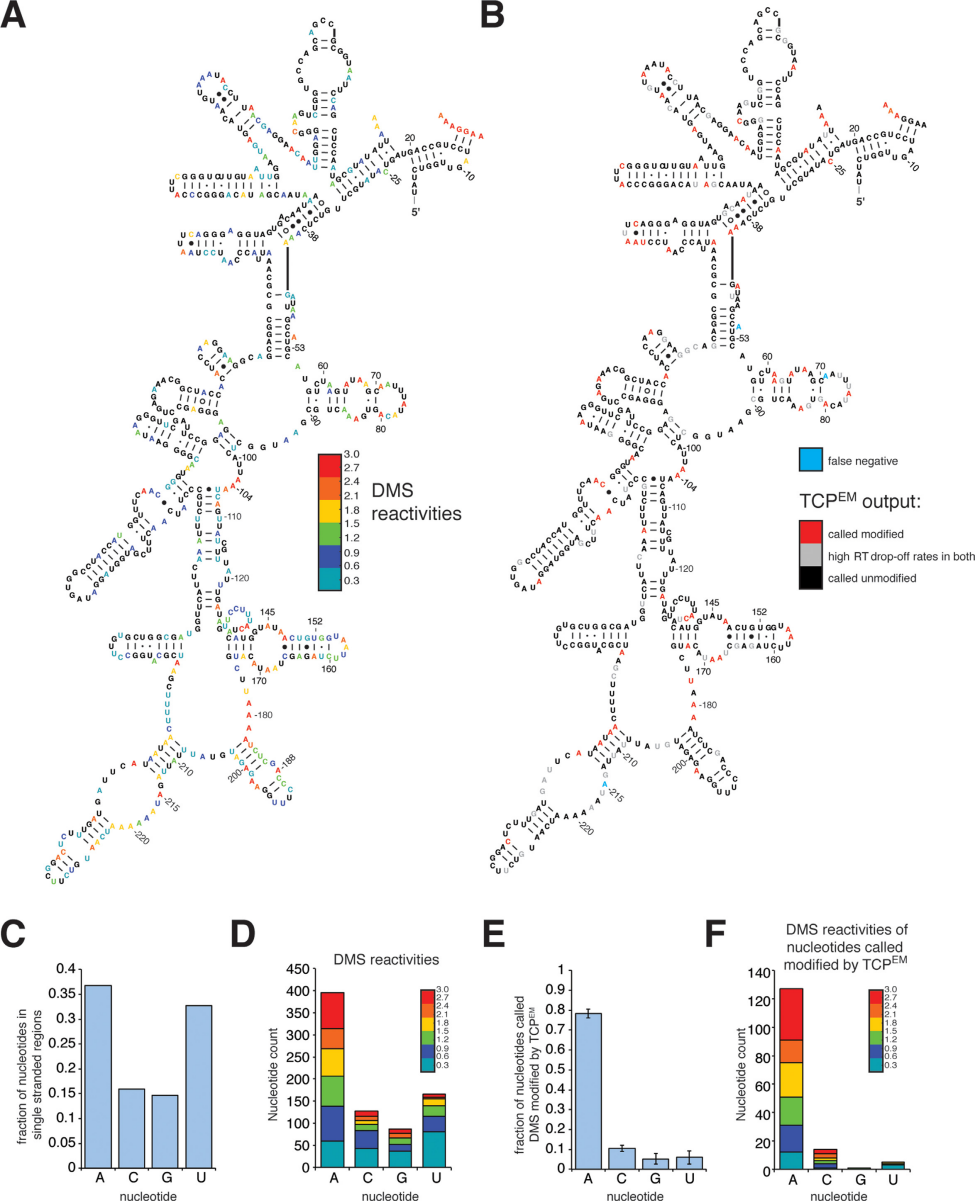
ChemModSeq accurately and quantitatively measures RNA secondary structure. (**A** and **B**) DMS reactivities (A) and TCP^EM^ outputs (B) for nucleotides in the 18S rRNA depicted in the secondary structure of the 5′ region of the yeast 18S rRNA. DMS reactivities were calculated as described (31) and capped at 3. Red colored letters in (B) indicate nucleotides called modified by the TCP^EM^ algorithm. Blue letters in (B) indicate nucleotides visually identified as modified by DMS in primer extension reactions (see Figure 2A) but not called modified by the TCP^EM^ algorithm. (**C** and **D**) Distribution of nucleotides located in single-stranded regions in the 18S rRNA (C) and the distribution of nucleotides with DMS reactivities higher than zero (D). (**E** and **F**) Fraction of nucleotides called DMS-modified by the TCP^EM^ algorithm (E; average and standard deviations from three independent experiments) and the DMS reactivities of these nucleotides (F). DMS reactivities were calculated by summing drop-off counts from three independent experiments.

For each nucleotide in the 18S rRNA we then calculated DMS reactivities (as described by (31); Figure 3A and D). Although adenosines generally had higher DMS reactivities, the data also indicated modification of a higher than expected number of guanines and uracils and the pattern was similar to the distribution of nucleotides in single-stranded regions (Figure 3C and D). We then applied the TCP^EM^ algorithm to the same data at a setting in which a nucleotide was only considered ‘modified’ if its probability of being modified was higher than 0.90 (Figure 3B and E). This greatly reduced the noise: 90% of the nucleotides called modified by the TCP^EM^ algorithm were adenosines and cytosines (compare Figure 3F with D), which is consistent with the known chemical reactivity of DMS (35). Contrary to DMS data generated using the Mod-seq protocol (33), our ChemModSeq data did not show significant modification of guanine residues (Figure 3B and E).

The majority of the nucleotides identified as modified in the 5′ end of the 18S rRNA were located in single-stranded regions (Figure 3B). Modification of base-paired nucleotides (Watson–Crick and non-Watson–Crick) at the base of stem structures indicated that these regions were flexible under our purification conditions. Comparison of the primer extension data (Figure 2A) with the TCP^EM^ output revealed that the number of false negatives was low (Figure 3B, blue colored nucleotides). Notably, the RT drop-off rates of the nucleotides called modified by the TCP^EM^ algorithm were uncorrelated with solvent accessibility of the DMS reactive groups in single-stranded regions (Supplementary Figure S3A). This emphasizes that DMS reactivity also does not simply provide a read-out of solvent accessibility (36).

We conclude that ChemModSeq quantitatively and accurately detect sites of chemical modification and, in combination with the TCP^EM^ algorithm, produces better signal-to-noise ratios than existing methods.

### Composition of pre-40S particles allows a classification into early, middle and late particles

**Figure 4.**
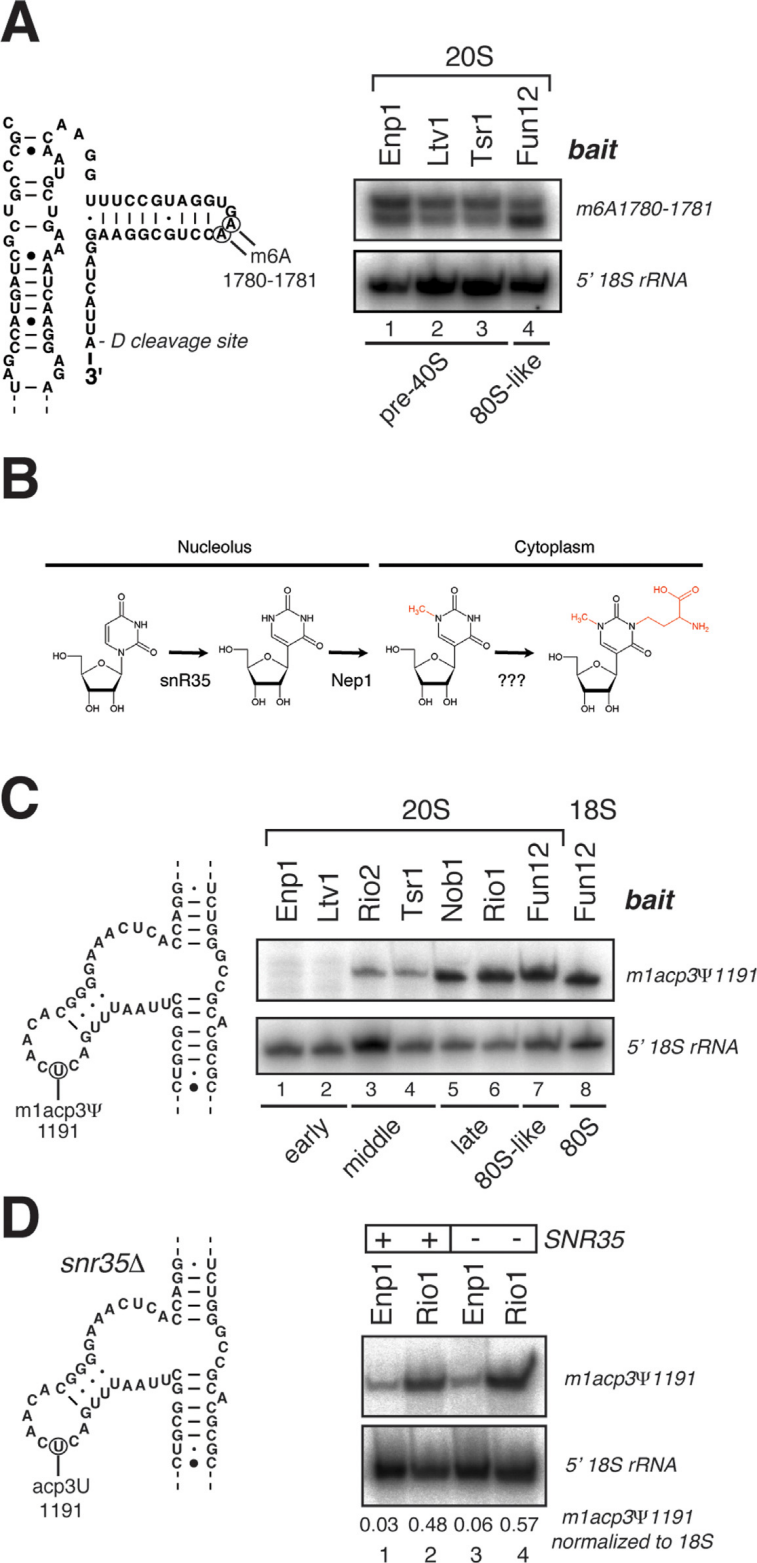
Pre-40S complexes can be classified into early, middle and late particles. (**A**) Detection of cytoplasmic methylation at A1780 and A1781 by primer extension in 20S pre-rRNAs. As loading controls, we performed primer extensions to detect the A1 cleavage site at the 5′ end of the pre-rRNAs. (**B**) Hypermodification of U1191 in the 18S rRNA. U1191 is first pseudouridinylated and N1-methylated by snR35 and Nep1 in the nucleolus, respectively, and then aminocarboxypropyl (acp)-modified in the cytoplasm by an unknown enzyme. (**C**) Detection of U1191 acp modification in 20S and 18S rRNAs by primer extension. Baits used to purify the individual pre-rRNA species are indicated above the panel. Loading controls were as in (A). (**D**) Same as in (C) but compared to strains lacking the *SNR35* gene.

We hypothesized that pre-40S complexes purified with the various baits (Figure 1C) might represent a mixture of particles at various maturation stages. To get an impression of their heterogeneity, we measured the level of Dim1-dependent cytoplasmic methylation at A1780 and A1781 in purified rRNAs by primer extension (Figure 4A). All 20S pre-rRNAs analyzed were methylated at these sites. We could, however, reproducibly see a stronger signal at A1781 in the 20S pre-rRNA associated with Fun12 in the late 80S-like complexes (also see below). This suggests that Dim1-dependent methylation at A1781 was not complete in the purified 20S pre-rRNAs associated with Ltv1, Enp1 and Tsr1. Hence, these baits likely purified a mixture of nuclear and cytoplasmic complexes. U1191 is acp-modified by an unknown enzyme just before D-site cleavage (3) (Figure 4B). Because this modification blocks RT (4), we measured the level of acp modification in purified rRNAs by primer extension. Intriguingly, this revealed that the level of acp modification at U1191 depended on the bait used for purifying the 20S pre-rRNA (Figure 4C). High RT drop-off signals at U1191 were found primarily in 20S pre-rRNA associated with Nob1, Rio1 and 80S-like particles, at levels comparable to the mature 18S rRNA (Figure 4C, lanes 5–8). In contrast, 20S pre-rRNAs associated with Ltv1 and Enp1 contained very low levels of U1191 acp modification (Figure 4C, lanes 1 and 2), whereas intermediate levels were detected in 20S associated with Rio2 and Tsr1 particles (Figure 4C, lanes 3 and 4). To rule out that other U1191 modifications significantly contributed to the primer extension signal, we performed primer extension analyses on 20S pre-rRNAs isolated from strains in which *SNR35* was deleted (Figure 4D). This deletion blocks U1191 pseudouridylation and Nep1-dependent N1 methylation but not acp modification (4,37) (Figure 4B). These experiments confirmed that the acp modification is primarily responsible for the strong stop observed in primer extension reactions. Based on these observations we categorized the pre-40S intermediates into early (Ltv1, Enp1), middle (Rio2, Tsr1) and late (Nob1, Rio1, 80S-like) particles.

### Late Rio1 pre-40S particles lack many 40S assembly factors

**Figure 5.**
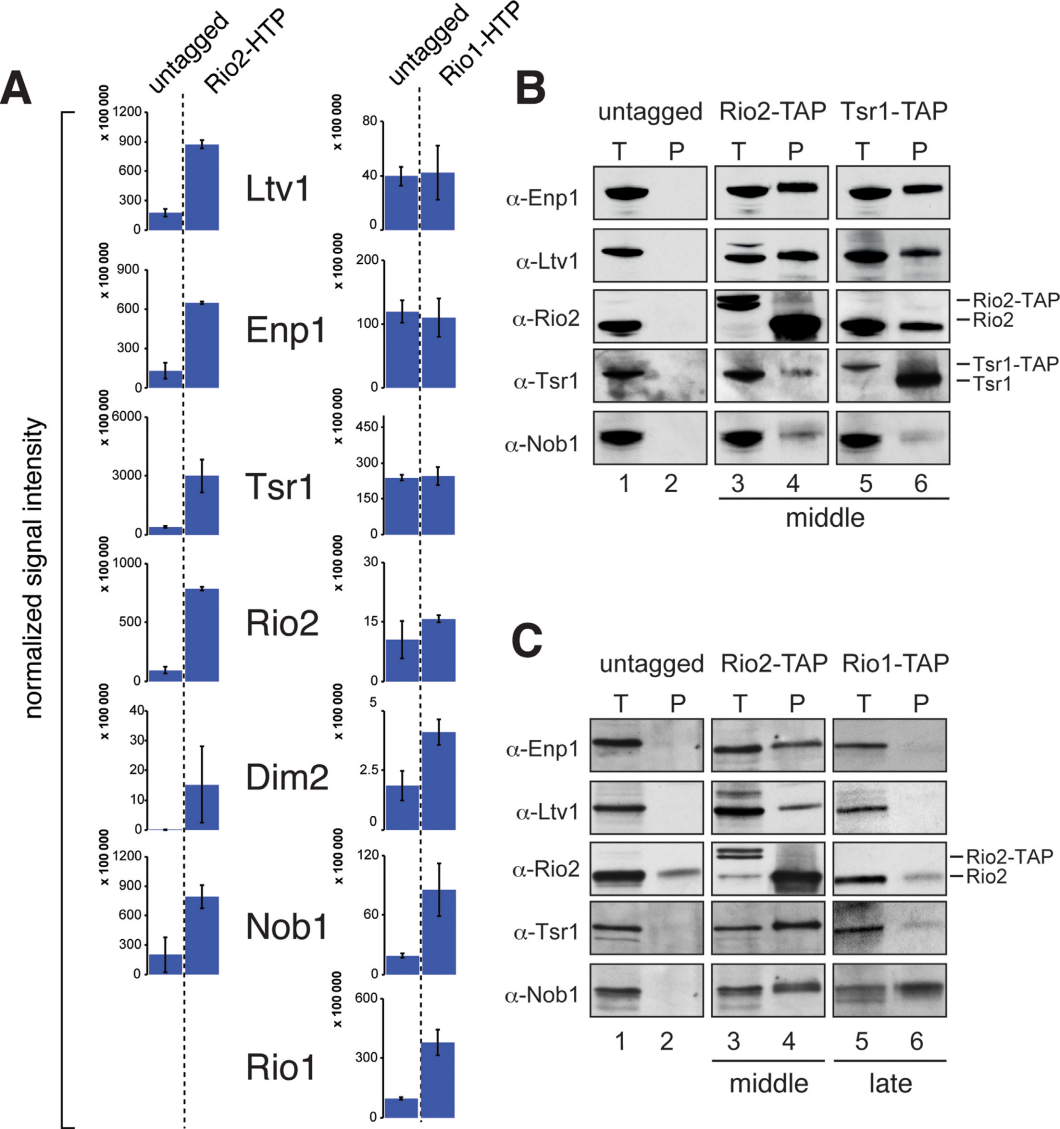
Rio1 associated pre-40S complexes contain fewer 40S assembly factors. (**A**) HTP-tagged Rio2 and Rio1 were affinity purified and associated proteins were quantified by label-free LC-MS. Shown are the averages and standard deviations from two (Rio2) or three (Rio1) replicate experiments. The y-axis indicates the relative abundance of the proteins in the purified complexes. The number of peptides used to calculate these abundances is listed in Supplementary Table S1. No Rio1 peptides were found in the Rio2 affinity purification. (**B** and **C**) Western blot analysis of protein composition of middle (Rio2, Tsr1) and late (Rio1) pre-40S particles purified by Tandem Affinity Purification (TAP) using TAP-tagged strains.

The remarkable variation in the level of U1191 acp modification in the purified 20S pre-rRNAs suggests that Enp1 and Ltv1 are probably largely absent in late pre-40S intermediates, whereas Rio2 and Tsr1 are present in sub-stoichiometric amounts. To test this hypothesis, we compared the protein composition of middle (Rio2, Tsr1) and late (Rio1) pre-40S particles. The HTP-tagged Rio1 protein, which we used as bait for all our structure probing experiments, only transiently interacted with pre-40S complexes (Supplementary Figure S4). Therefore, to detect Rio1 associated proteins, we had to use a fast IgG affinity purification protocol (38) followed by label-free quantitative mass spectrometry. These results were then compared to mass spectrometry data obtained using a Rio2-HTP strain. This revealed that Rio1-HTP only significantly enriched for Nob1 and Dim2 (Figure 5A). Rio2 efficiently co-precipitated all of the assembly factors analyzed, except for Rio1, which was not detected in Rio2-HTP purifications (Figure 5A). This suggests that these two proteins are most likely not present in the same complex. To confirm that Rio1 interacts with Nob1 in pre-40S complexes, we performed tandem affinity purifications with strains expressing TAP-tagged versions of Rio2, Tsr1 and Rio1 (Figure 5B and C). Contrary to Rio1-HTP, the TAP-tagged Rio1 protein co-sedimented almost exclusively with 40S-sized particles in sucrose gradients, suggesting it is stably bound to pre-40S complexes (Supplementary Figure S4). Western blot analyses showed that TAP-tagged Rio2 and Tsr1 efficiently co-precipitated all of the tested factors (Figure 5B, lanes 4 and 6), indicating that Rio2 and Tsr1 ‘middle’ particles share most of the assembly factors. Consistent with our mass spectrometry analyses, TAP-tagged Rio1 only significantly enriched for Nob1 (Figure 5C, lane 6).

We conclude that, as in human cells (13), late Rio1 pre-40S complexes lack the majority of 40S assembly factors.

### ChemModSeq reveals RNA structural differences between early-middle and late pre-40S particles

**Figure 6.**
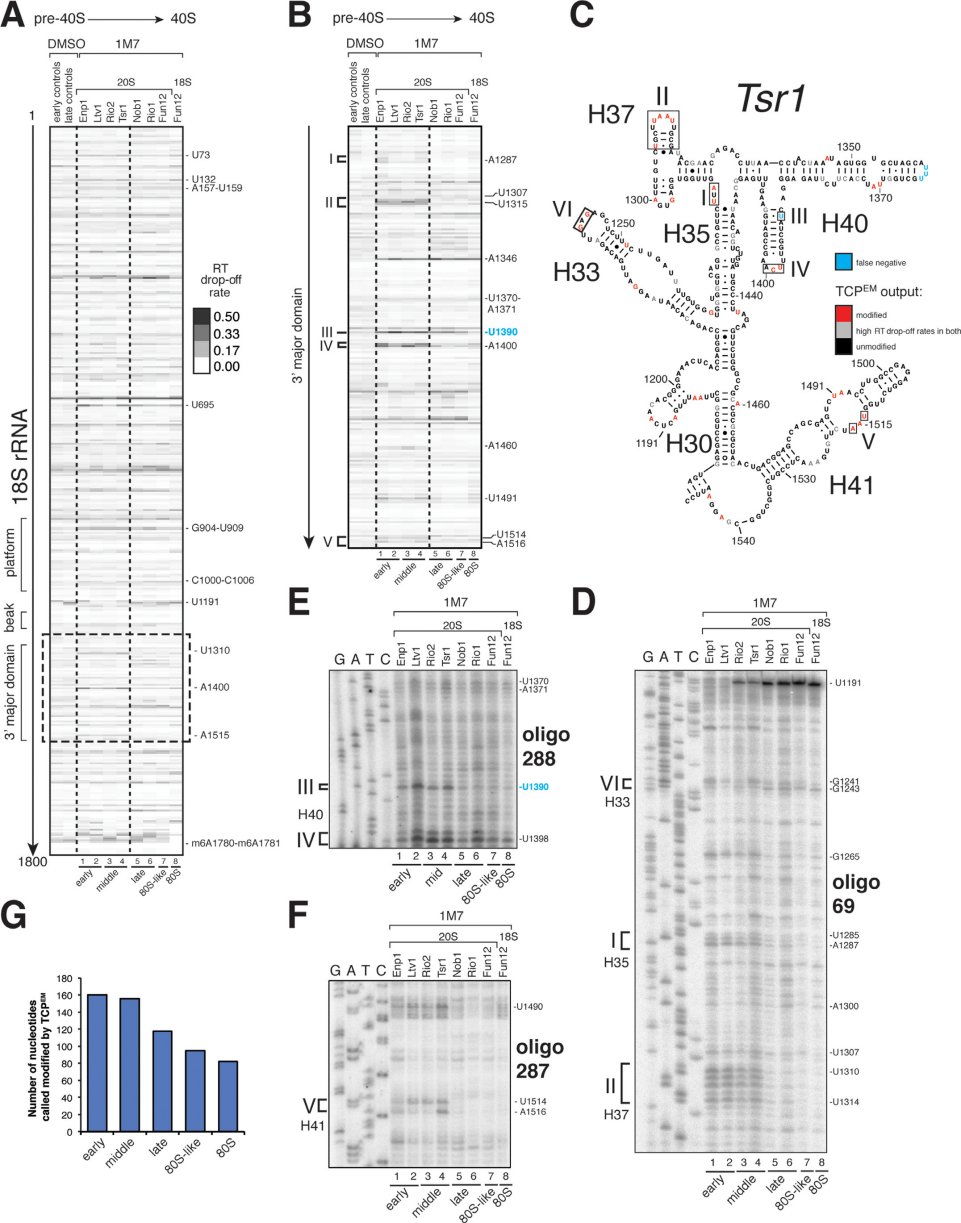
A digital snapshot of pre-rRNA secondary structures. (**A** and **B**) ChemModSeq and primer extension results obtained using rRNAs extracted from *in vitro* 1M7 modified particles. Heat maps of average RT drop-off rates in 18S coding sequences (*n* ≥ 2) (A) or the 3′ major domain (B) of purified rRNAs. The positions of the modified nucleotides are indicated on the right side of each panel. (**C**) Early-middle and late pre-40S particles are structurally distinct. The 3′ major domain secondary structure represents the ChemModSeq results for the middle (Tsr1) particle. Red nucleotides were indicated as modified by the TCP^EM^ algorithm. Blue nucleotides were visibly modified by 1M7 but not called modified by the TCP^EM^ algorithm (false negative). (**D**–**F**) Representative primer extension reactions for the 3′ major domain. Roman numbers indicate sites where differences in SHAPE reactivity were observed between pre-40S particles. Oligonucleotides used for primer extensions are indicated in bold. The positions of the modified nucleotides are indicated on the right side of each panel. (**G**) Pre-rRNAs in early and middle pre-40S complexes adopt more flexible conformations compared to late particles. Plotted are the average number of nucleotides called modified by the TCP^EM^ algorithm (y-axis) for early, middle, late, 80S-like and mature 80S translation initiation complexes (x-axis).

We next applied our ChemModSeq protocol to modified rRNAs purified from 40S assembly intermediates. The SHAPE reagent 1M7 was the preferred chemical probe for these experiments as it generically reacts with 2′-OH in all four nucleotides in single-stranded and flexible regions (14) and therefore would yield more structural information than DMS. Figure 6A and B show heat map representations of average RT drop-off rates for all samples for the nucleotides in the 18S rRNA and the 3′ major domain, respectively. Drop-off rates for the 18S rRNA for all samples are shown in Supplementary Table S2. Results for Internal Transcribed Spacer 1 (ITS1) will be described elsewhere (manuscript in preparation).

In agreement with the primer extension results shown in Figure 4, average drop-off rates at U1191 in unmodified 20S pre-rRNA samples were consistently higher in late particles, and high drop-off rates could be detected at the Dim1-dependent methylation sites in all samples (Supplementary Figure S5A and B).

The heat maps shown in Figure 6A and B revealed clear differences in nucleotide reactivity to 1M7 between early, middle and late particles (see regions highlighted with roman numbers). We then applied the TCP^EM^ algorithm to identify regions where statistically significant differences in chemical reactivity could be detected between RNA samples. The TCP^EM^ algorithm greatly simplified the interpretation of the data as it allowed us to identify easily rRNA regions that had the highest likelihood of being restructured during 40S assembly (Figure 6C; highlighted with boxes and roman numbers).

To validate the TCP^EM^ results, we performed primer extension analyses (Figure 6D–F). This confirmed the observed differences in SHAPE reactivity between the various purified RNAs (see regions highlighted with roman numbers in Figure 6D–F). Bioinformatics analyses of the 18S rRNA SHAPE ChemModSeq sequencing data revealed that RT drop-off rates of nucleotides identified as modified by the TCP^EM^ algorithm was uncorrelated with 2′OH solvent accessibility of single-stranded regions (Supplementary Figure S3B and C), reinforcing the observation that SHAPE chemicals provide a read-out of RNA dynamics, not RNA accessibility (15,20).

Generally, a larger number of nucleotides were modified in early and middle assembly intermediates suggesting that these adopt more flexible rRNA conformations (Figure 6G). The 3′ major domain in particular was much more reactive to 1M7 in early and middle particles. These included nucleotides in H33, H35, H37, H40 and H41 (Figure 6B–F, boxed regions indicated with roman numbers). 1M7 modified 18S isolated from empty and salt-washed free 40S particles (see Supplementary Methods), did not show the same degree of flexibility in H35, H37, H40 and H41 (Supplementary Figure S6). This indicated that many of the observed changes in SHAPE reactivity in the 3′ major domain were not the result of pre-40S complexes joining with 60S subunits or binding of tRNA to the decoding center.

Just before we completed the *in vitro* 1M7 experiments, a new SHAPE reagent NAI for probing of RNA secondary structures in living cells was reported (21). To substantiate our results we compared middle (Tsr1) and late (Rio1) with mature (Fun12) particles modified *in vivo* using NAI (Supplementary Figure S7). Importantly, the same regions in the 3′ major domain were also modified by NAI in early and middle pre-40S complexes *in vivo*, indicating that the observed differences in 1M7 reactivity were not the result of r-proteins dissociating during the purification (Supplementary Figure S7, boxed regions in gels and secondary structure model). We note that some nucleotides reacted differentially to NAI and 1M7. Our *in vitro* 1M7 probing experiments were performed using MgCl_2_ concentrations that support translation. Under these conditions ribosomal subunits are known to have higher conformational dynamics, which may account for some of the differences observed (39). We can also not rule out the possibility that pre-rRNAs undergo slight structural changes during the purification procedure. Finally, some SHAPE chemicals also have different preferences for nucleotide conformations (40).

We conclude that the 3′ major domain in the 20S pre-rRNA is restructured during late stages of maturation.

### Restructuring of the 3′ major domain coincides with the release of assembly factors

**Figure 7.**
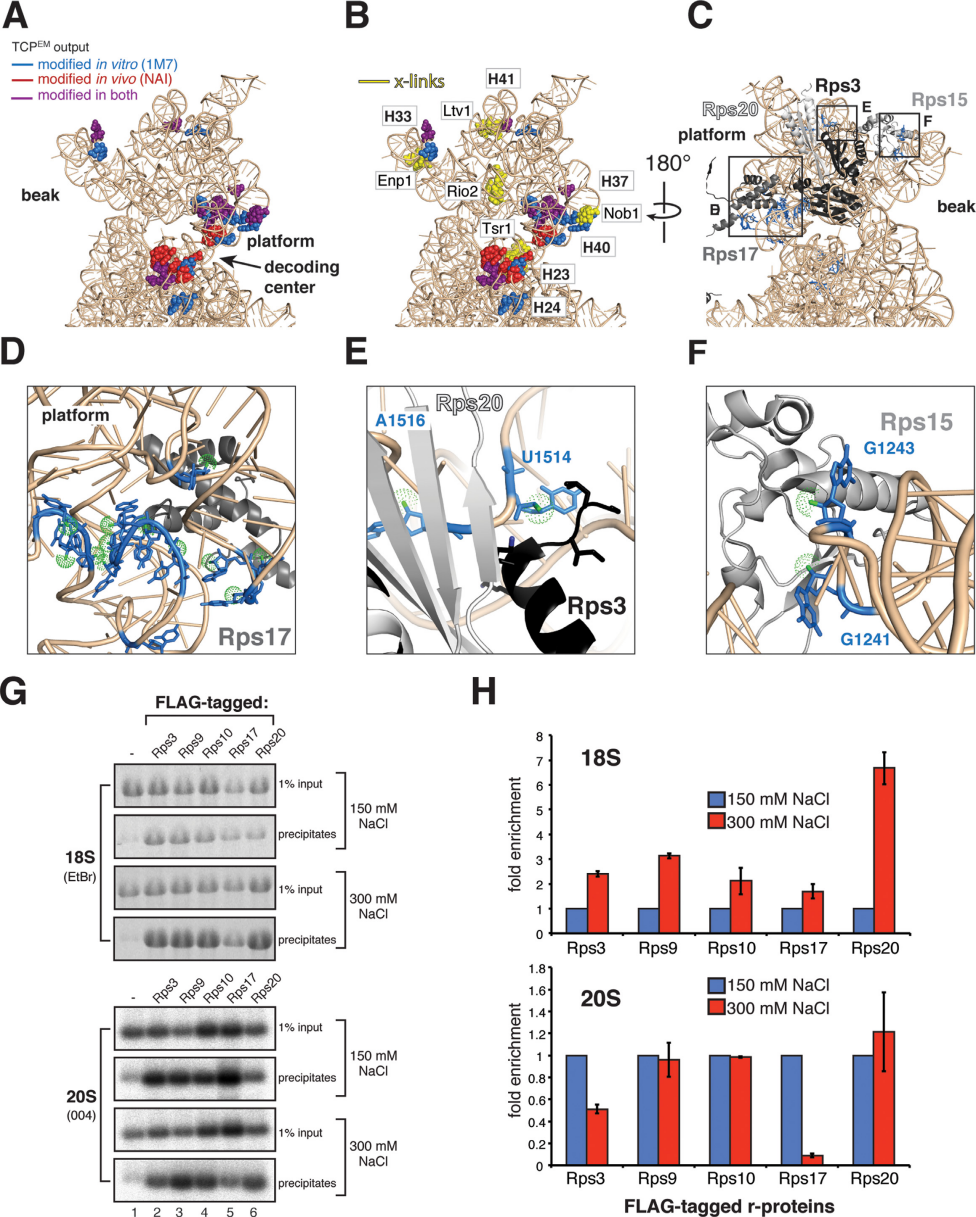
Ribosomal protein rRNA binding sites are highly flexible in early and middle pre-40S complexes. (**A** and **B**) Nucleotides in the head domain reactive to SHAPE chemicals (A) cluster near assembly factor UV cross-linking sites (B). (**C**–**F**) SHAPE-modified nucleotides (blue) in the head domain coincide with Rps3, Rps15, Rps17 and Rps20 binding sites. Relevant nucleotide positions are indicated. Green dots highlight the 2′-OH. (**G** and **H**) Ribosomal proteins Rps3 and Rps17 form salt-labile interactions with pre-40S complexes. Immunoprecipitations were performed using strains expressing FLAG-tagged r-proteins (indicated above the panels). Co-precipitated 20S was detected by northern hybridization with oligo 004, 18S rRNA was detected by ethidium bromide staining (EtBr). Input indicates 1% of total RNA extracted from cell lysates. (H) Quantification of results shown in (G). Error bars indicate standard deviations obtained from two biological replicates.

Many of the highly flexible nucleotides in early-middle pre-40S complexes clustered near previously identified assembly factor UV cross-linking sites (see (5)) (Figure 7A and B). This indicates that these factors play a role in maintaining a more flexible rRNA conformation. In late pre-40S particles, including the Rio1 particle, the 3′ major domain was significantly less reactive to SHAPE chemicals (Figure 6B–D, Supplementary Figure S7A–E, regions indicated with roman numbers). The observed structural rearrangements in the 3′ major domain therefore presumably occur before Rio1 enters the assembly pathway and may coincide with the release of assembly factors Rio2, Tsr1, Ltv1 and Enp1. Although Nob1 assembles into pre-ribosomes at a very early stage (41), and cross-links to the 3′ major domain (5), it does not appear to contribute to these RNA restructuring events, as pre-40S complexes that accumulated in cells lacking Nob1 had all the hallmarks of late pre-40S particles (Supplementary Figure S8: see regions indicated with roman numbers).

### Ribosome assembly factors are required to maintain an rRNA conformation that is incompatible with stable binding of Rps17 and Rps3

Several of the highly flexible nucleotides detected in early-middle pre-40S particles are contacted by r-proteins in the mature subunit (42). For example, the terminal loop of H37 and the stem of H40 are contacted by Rps17 (Figure 7D). Nucleotides U1514 and A1516, located in an internal loop in H41 in close proximity to an Ltv1 cross-linking site, contact Rps20 (A1516) and the N-terminus of Rps3 (U1514), (Figure 7E). Thus, although these r-proteins are present in pre-40S particles (43) they might not contact these sites in early and middle pre-40S complexes. If so, then it might be expected that these r-proteins are less stably associated with pre-40S particles compared to mature subunits. Consistent with this idea, Rps3 dissociates when pre-40S particles are subjected to high salt conditions (7). To test whether this is also the case for other r-proteins, we performed immunoprecipitation experiments with strains expressing FLAG-tagged r-proteins (Figure 7G and H). Under high salt conditions the binding of both Rps3 and Rps17 to pre-40S complexes was reduced, whereas their recovery with mature 40S subunits was more efficient than under low salt conditions. This indicates that these r-proteins are recruited to pre-ribosomes, but are not in their final conformation in early-middle pre-ribosomes.

Notably, our data also revealed rRNA structural rearrangements in the beak. In mature 40S subunits, Rps15 binds to G1241 in the tip of H33 (Figure 6C, box VI) close to an Enp1 cross-linking site (Figure 7B). G1243, which is exposed to the surface in the crystal structure (Figure 7F), reacted more strongly with SHAPE chemicals in late pre-40S and mature 40S particles. G1241, however, was more reactive to 1M7 in early intermediates (Figure 6D). This indicates that Rps15 may only interact with the tip of H33 once the beak has been formed.

**Figure 8.**
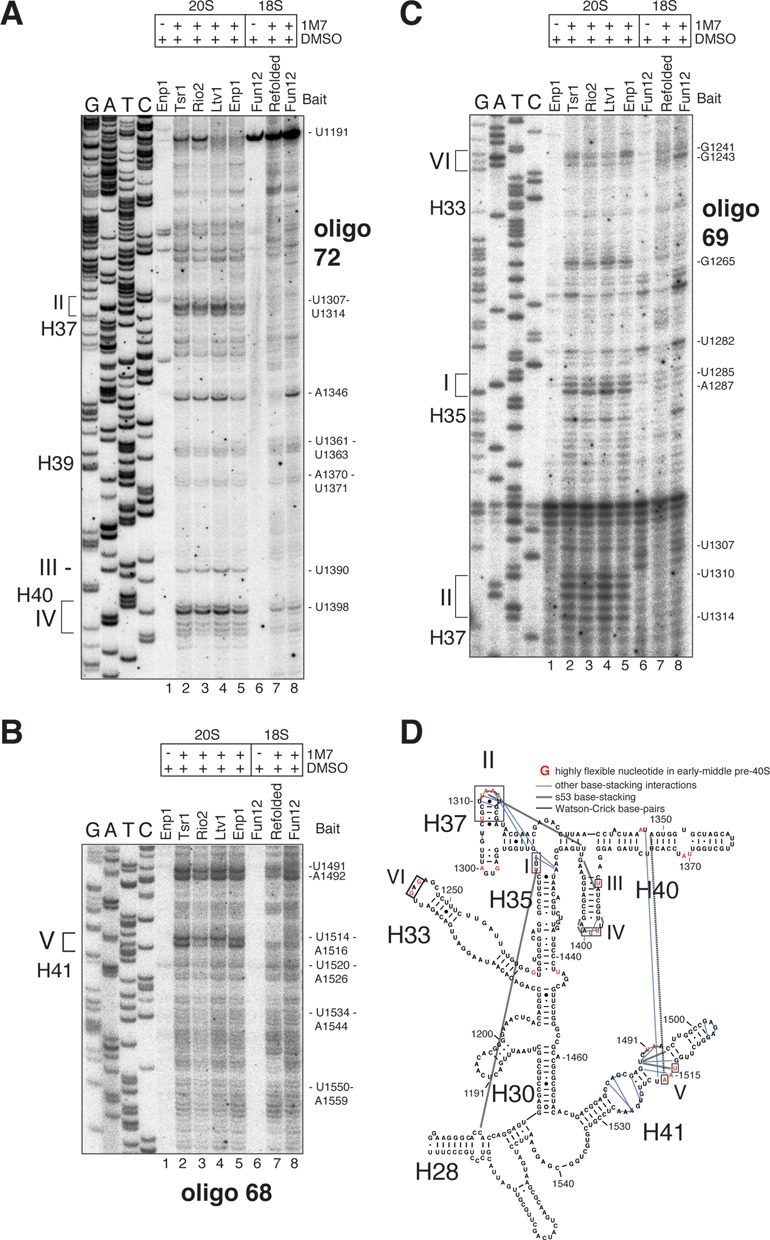
Ribosome assembly factors are required to maintain an open head domain conformation. (**A**–**C**) Comparison of primer extension data obtained from 1M7 modified early (Ltv1, Enp1), middle (Tsr1, Rio2) pre-40S complexes (lanes 2–5), mature 40S subunits (Fun12) (lane 8) and *in vitro* refolded 18S rRNA (lane 7, Refolded). Unmodified 20S and 18S rRNAs were used as control samples (lanes 1 and 6). The positions of the modified nucleotides are indicated on the right side of each panel. (**D**) Overview of TCP^EM^ output generated from *in vitro* refolded 18S rRNA ChemModSeq data. Shown are the results for the 3′ major domain. Yellow letters indicate nucleotides that the algorithm predicted were most likely 1M7 modified.

To address whether the observed changes in nucleotide flexibility between early-middle and late pre-40S particles could be the result of ribosomal protein binding events, we performed 1M7 probing experiments on 18S rRNA that was *in vitro* refolded under the same conditions used to purify pre-40S complexes (Figure 8A–C, compare lanes 7 and 8). Comparison of the SHAPE reactivity profiles revealed that large regions of the 3′ major domain in the refolded rRNA adopted secondary structures similar to 18S and 20S rRNA molecules in purified particles (Figure 8A–C). Surprisingly, the majority of the highly SHAPE reactive nucleotides in early and middle pre-40S particles that clustered near assembly factor binding sites (H33, H35, H37, H40 and H41; see Figure 7) did not show the same degree of flexibility in *in vitro* refolded RNA (Figure 8D, red colored nucleotides in regions indicated with roman numbers). This suggests that under the conditions used, these regions can fold into, what appear to be, relatively stable structures independently of proteins *in vitro*. In the yeast 80S crystal structure, the nucleotides in the H37 terminal loop and the internal loop in H41 (U1514–U1517) that do not form Watson–Crick base-pairs, form a large network of base-stacking interactions, including many long-range interactions (Figure 8D, indicated with dotted lines). These presumably help stabilize the structure of the rRNA. Many of these stacking interactions are s53 interactions (5′ side of one base with 3′ side of other base) that are generally less reactive to SHAPE reagents (44), The relatively low SHAPE reactivity of these nucleotides in the *in vitro* refolded 18S rRNA suggests that at least some of these base-stacking interactions form in the absence of proteins. Based on these results, we hypothesize that assembly factors that associate with early and middle pre-40S particles function by providing the energy necessary to maintain a flexible 3′ major domain conformation.

## DISCUSSION

Ribosome synthesis in eukaryotes is a highly complex and dynamic process that involves ordered assembly of r-proteins and numerous RNA structural rearrangements. Hundreds of assembly factors are also involved in the process, many of which have the capacity to extract energy from nucleotide tri-phosphates and presumably play key roles in these restructuring events. Major challenges in the ribosome synthesis field have been to find methods that reveal detailed mechanistic insights into the assembly pathway and to assign functions to assembly factors. Because the assembly intermediates are heterogeneous and dynamic, they are difficult to characterize structurally and other complementary approaches are needed to address these mechanistic questions. Inspired by impressive chemical probing work done by many groups on bacterial ribosomes, we have developed protocols for purification and chemical modification of specific yeast ribosome assembly intermediates. By combining SHAPE chemical probing with high-throughput sequencing and statistical modeling, we were able to rapidly and quantitatively measure RNA structural changes that take place during 40S ribosome synthesis in yeast. Our method, dubbed ChemModSeq, provides the first digital snapshots of pre-rRNA nucleotide dynamics during 40S assembly, revealing new mechanistic insights into pre-rRNA restructuring events and the function of 40S assembly factors. To detect sites of modification with high accuracy, we developed a two-channel Poisson EM algorithm (TCP^EM^). This algorithm calculates for each nucleotide the likelihood that it is chemically modified, allowing the detection of sites where statistically significant differences in chemical reactivity between various RNA samples could be identified. We demonstrate that the algorithm can greatly reduce noise in structure probing sequencing data, increasing the reliability of the results. TCP^EM^ also simplified the interpretation of the data as it allowed us to quickly identify regions that were most likely restructured during 40S assembly.

The ChemModSeq protocol and the statistical methods described here are applicable to any ribonucleoprotein complex.

### 40S assembly factors chaperone pre-rRNA folding steps during 40S assembly

Previous work had indicated that the binding sites of several 40S ribosome assembly factors is incompatible with translation initiation, as many occupied tRNA and translation initiation factor binding sites (5,6). It was proposed that these factors play an important role in quality control by blocking premature formation of translation initiation complexes (6). An alternative, but not mutually exclusive, interpretation of these results is that binding of ribosome assembly factors to functionally important regions is required for the formation of the functional center of the ribosome (5,45). In agreement with this model, our results suggest that ribosome assembly factors play an important role in regulating RNA folding events that take place during late stages of 40S assembly.

**Figure 9.**
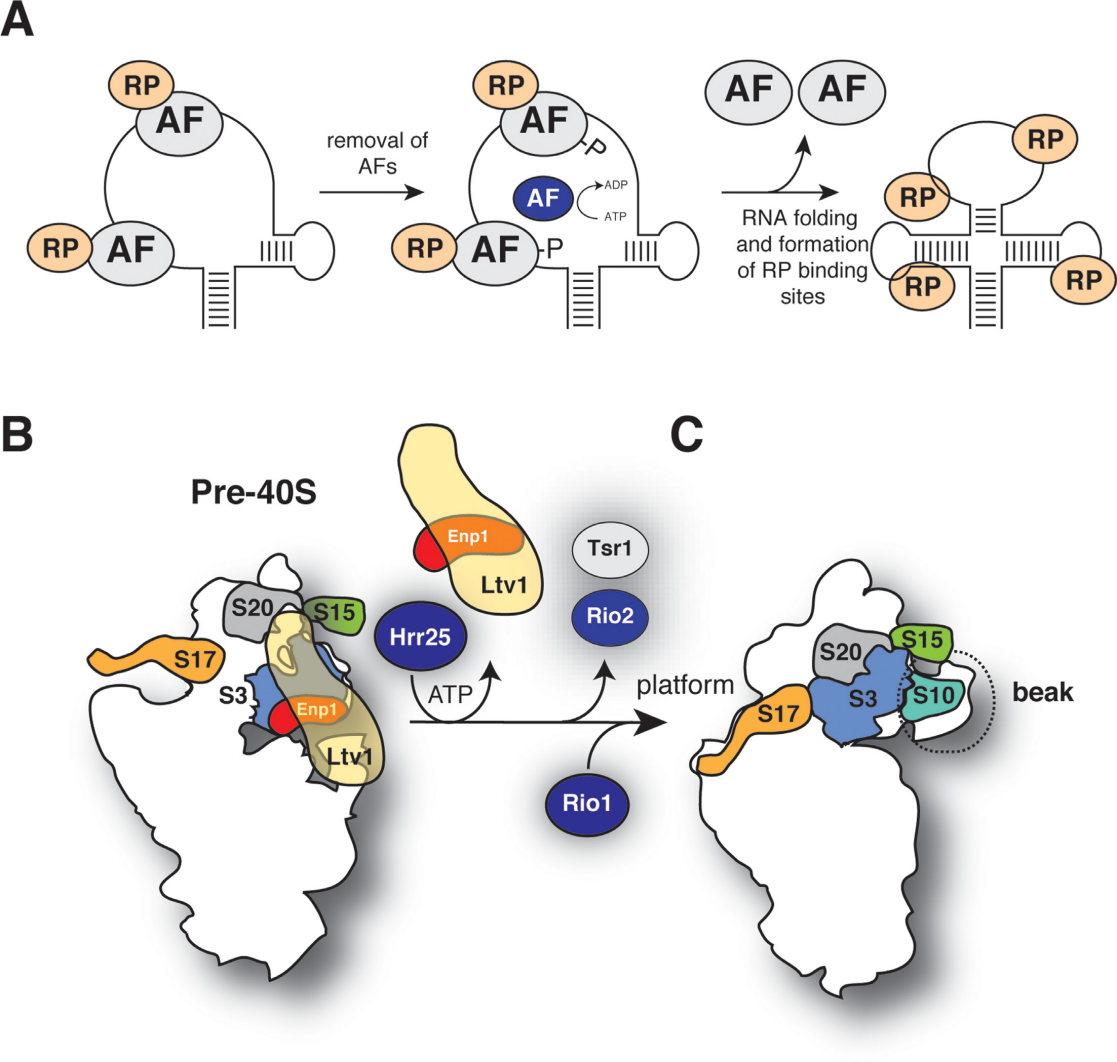
A model for structural rearrangements during late stages of 40S subunit maturation. (**A**) Proposed model for the function of ribosome assembly factors in 40S assembly. Binding of assembly factors (AF) to RNA is required to maintain a more flexible conformation. Their presence allows the assembly of ribosomal proteins (RP) but prevent that these RPs adopt their final conformation. At some point during the maturation pathway, the system receives a signal that certain ribosome assembly/RNA folding steps have been completed and AFs are no longer needed. Their release could be triggered through phosphorylation (such as Hrr25-dependent phosphorylation of Enp1 and Ltv1). This allows completion of RNA folding steps and binding of late RPs. (**B** and **C**) Model for rearrangements that take place in the head domain during late stages of 40S assembly. In early and middle pre-40S complexes, binding of Ltv1 and Enp1 to the head domain is required to maintain a more flexible conformation in the 3′ major domain. Rps3, Rps17, Rps20 and Rps17 assemble into pre-ribosomes but do not adopt their final conformation. Hrr25 phosphorylation of Ltv1 and Enp1 triggers their release, allowing late RNA folding steps and assembly of head domain r-proteins to be completed. We propose that Rio2 and Tsr1 are released shortly afterward (B) and Rio1 enters the assembly pathway. The exact function of Rio1, however, remains unclear.

The ChemModSeq analyses revealed distinct differences in the pattern of nucleotide SHAPE reactivity between 40S assembly intermediates. Pre-40S particles that contained Rio2, Tsr1, Ltv1 and Enp1 were generally more flexible compared to late pre-40S complexes that lacked these proteins. Many of these flexible nucleotides in early-middle pre-40S complexes clustered near assembly factor binding sites, including Ltv1 and Enp1 binding sites in the 3′ major domain. Interestingly, our ChemModSeq analyses on *in vitro* refolded 18S rRNA (Figure 8) demonstrated that many of these highly flexible regions could spontaneously fold into, what appeared to be, relatively stable structures. We propose that assembly factors function by providing the necessary energy to maintain a more flexible conformation in the pre-rRNA (Figure 9A). More specifically, we hypothesize that Ltv1 and Enp1, which bind to the head domain, act as RNA chaperones by delaying folding steps in the 3′ major domain (Figure 9A). This would be required to allow earlier pre-rRNA folding steps to be completed and/or to safeguard correct folding by preventing the accumulation of kinetically trapped intermediates. A similar function has been proposed for bacterial RimM, which regulates 3′ major domain formation by holding the rRNA in a conformation that allows proper folding of the beak structure and H43 (46). Similarly, the U3 and snR30 snoRNAs regulate the formation of long-range rRNA interactions in the 18S rRNA. The U3-rRNA-5′18S rRNA base-pairing interaction blocks the formation of the universally conserved central pseudoknot (47), whereas snR30-rRNA base-pairing interferes with long-range interactions between two rRNA expansion segments (48). At some point during the maturation pathway, certain assembly factors are no longer required and need to be released. This can be achieved through phosphorylation of proteins (such as Hrr25-dependent phosphorylation of Enp1 and Ltv1, Figure 9B; (7)), which presumably reduces their affinity for RNA, or through the activity of other energy-dependent remodelers, such as RNA helicases and GTPases (e.g. see (49,50)). Release of these factors would allow completion of RNA folding steps, trigger binding of late assembling r-proteins (our work) or trigger remodeling steps (7,51).

We and others have shown that the presence of assembly factors affects stable binding of Rps17 and Rps3 (Figure 7 and (7)). Several of the more flexible nucleotides in H37 and H40 detected in early and middle pre-40S particles are contacted by Rps17 in the mature subunit (42). Rps17 is present in pre-40S particles (43); however, our data suggest that Rps17 may not contact these sites during early stages of 40S maturation. Our results also indicate that the interaction between Rps15 and the tip of H33 may only take place at late stages of assembly once the beak has been formed (Figure 9B).

It is not entirely clear why some r-proteins do not adopt their final conformation in early-middle pre-40S complexes. Plausible explanations are that this is necessary to maintain the more flexible conformations in 20S pre-rRNA observed in pre-40S complexes, to prevent non-specific protein–RNA and/or ensure that binding sites for late assembling r-proteins remain accessible. It has also been suggested that a flexible head domain conformation is important for efficient nuclear export (7). We note that both Rps3 and Rps15 are required for efficient nuclear export of pre-40S complexes (52,53) and it is possible that their assembly state is a measure of export competence. Similarly, 60S subunit assembly also seems to involve gradual strengthening of r-protein binding to pre-ribosomes (54,55). The binding strength of many 60S r-proteins was shown to increase once internal transcribed spacer 2 (ITS2) is been removed (55). It is becoming clear that a major function of ribosome assembly factors is to direct the timely incorporation of r-proteins by modulating the rRNA folding state.

Several lines of evidence indicate that Ltv1 and Enp1 also play a role in recruiting Rps3, Rps15 and Rps20 to pre-ribosomes: Ltv1, Enp1 and Rps3 form a salt-resistant complex *in vitro* (7), Ltv1 binds Rps3 in the yeast two-hybrid assay (56) and binds Rps15 *in vitro* (57). Furthermore, Rps3 and Rps20 could not be detected in pre-40S complexes purified from cells lacking Ltv1 (6).

### Restructuring of the 3′ major domain coincides with the release of the majority of pre-40S assembly factors and takes place before Rio1 enters the assembly pathway

Our results suggest that restructuring of the 3′ major domain coincides with the release of Rio2, Tsr1, Ltv1 and Enp1, before Rio1 enters the assembly pathway (Figure 9B and C). This is based on the following observations: (i) The SHAPE reactivity profile of Rio1 associated 20S pre-rRNA and (ii) the level of acp modification were both very similar to what we observed with mature 18S rRNA (Figure 6). This implies that Rio1 primarily associates with late pre-40S complexes. (iii) Rio1 was not significantly enriched in Rio2-associated particles, indicating they are not present in the same complex (Figure 5) and (iv) immunoprecipitation experiments with epitope-tagged Rio1 showed only significant enrichment of Nob1 (Figure 5), consistent with recently published work (58). (v) Finally, data from the Tollervey lab confirmed that Rio1-associated pre-40S particles and the later 80S-like complexes mostly contain Dim2 and Nob1 (David Tollervey, personal communication). Hence, all the available data suggest that Rio1 associates with pre-40S particles that are in their final stages of maturation. We speculate that once Rio2, Tsr1, Ltv1 and Enp1 are released, r-proteins in the head domain can adopt their final conformation and late assembling r-proteins (such as Rps10) can assemble into pre-ribosomes (Figure 9B and C). The resulting pre-40S, containing Nob1 and Dim2 and possibly also Rio1, then joins with a 60S subunit to form an 80S-like complex. Successful joining may trigger Nob1-dependent cleavage at site D (10–12).

## ACCESSION NUMBER

Raw (fastq) and processed sequencing data can be downloaded from the NCBI Gene Expression Omnibus repository (http://www.ncbi.nlm.nih.gov/geo, accession number GSE52878).

## SUPPLEMENTARY DATA

Supplementary Data are available at NAR Online.

SUPPLEMENTARY DATA
