# Supplementary material for: Snapshots of pre-rRNA structural flexibility reveal eukaryotic 40S assembly dynamics at nucleotide resolution
Source: Nucleic Acids Res. 2014 Sep 8;42(19):12138–54. doi: 10.1093/nar/gku815 (PMC4231735; doi:10.1093/nar/gku815)
Supplement: SUPPLEMENTARY DATA [file supp_42_19_12138__index.html]

Snapshots of pre-rRNA structural flexibility reveal eukaryotic 40S assembly dynamics at nucleotide resolution — Snapshots of pre-rRNA structural flexibility reveal eukaryotic 40S assembly dynamics at nucleotide resolution — SUPPLEMENTARY DATA 

# Snapshots of pre-rRNA structural flexibility reveal eukaryotic 40S assembly dynamics at nucleotide resolution

## SUPPLEMENTARY DATA

**Files in this Data Supplement:**

- SUPPLEMENTARY DATA
- SUPPLEMENTARY DATA
