# Supplementary material for: Snapshots of pre-rRNA structural flexibility reveal eukaryotic 40S assembly dynamics at nucleotide resolution
Source: Nucleic Acids Res. 2014 Sep 8;42(19):12138–54. doi: 10.1093/nar/gku815 (PMC4231735; doi:10.1093/nar/gku815)
Supplement: SUPPLEMENTARY DATA [file supp_gku815_nar-01411-r-2014-File012.pdf]

SUPPLEMENTARY DATA

RNA extractions

RNA was extracted using a GTC phenol solution (4 M guanidine thiocyanate, 0.05 M Tris at pH 8.0, 0.01 M EDTA, 2% sarcosyl, 1% β-mercaptoethanol, 50% [v/v] phenol) as previously described (1, 2).

Primer extension reactions

Reverse-transcription reactions were performed using 1 pmol of ^{32}P-labeled oligonucleotide (Supplementary Table S4), 12 fmol of purified rRNA and Superscript III (Invitrogen) in a final volume of 5 µl, according to the manufacturer's instructions. Sequencing ladders were prepared using a plasmid containing an rDNA gene (3) and Sequenase v2.0 (USB/Affymetrix) according to the manufacturer's instructions. Samples were resolved on 6% PAA/8M Urea-TBE gels and visualized by autoradiography using the FUJI FLA5100 system.

Northern Blot analyses

Northern blots were carried out as previously described (4). Briefly, RNAs was resolved on 1.25% agarose gels and transferred to Hybond N^+ (Amersham). Hybridisation was performed in oligohyb buffer (0.3 M Na_2HPO_4, 0.15 M NaH_2PO_4, 0.25 M SDS, 10 mM EDTA), for 4-16 h at 37°C, using oligonucleotides listed in Supplementary Table S4. Membranes were washed with 6 x SSC at room temperature for 10 min followed by two 10 min washes with 2 x SSC, 0.1% SDS.

Sucrose density gradient analyses

Cells were grown to an OD_{600} of ~0.5 and lysed in TMN50 buffer (50 mM Tris pH 7.8, 50 mM NaCl, 1.5 mM $MgCl_2$, 0.2% Triton-X100 (v/v), 5 mM β-mercaptoethanol). Extract prepared from ~20 OD_{600} units of cells was loaded on a 13 ml 10-50% Sucrose gradient made in TMN50 and spun for 7 hours in a SW-40Ti rotor at 115,000 x g. Twenty 600 µl fractions were manually collected. RNA extractions and northern blot analyses were

performed as previously described (5). For the western blot analyses, proteins were TCA precipitated from 250 µl of gradient fractions and resolved on 4-12% Bis-Tris NuPAGE gels (Invitrogen) and transferred to nitrocellulose membranes. Antibodies used were: Peroxidase anti-peroxidase (PAP; Sigma; 1:5000), anti-TAP (Pierce; 1:5000), anti-S14 (Cambridge BioScience; 1:2000) and goat-anti-rabbit (Pierce; 1:10.000).

Tandem affinity purifications

For tandem-affinity purifications (TAP), cells were harvested at OD_{600} ~1.0 and lysed in TMN150 buffer as described above. Lysates were incubated with 250 µl of IgG Sepharose beads (Amersham) for one hour at 4ºC. After washing the beads three times with 10 ml of TMN150, complexes were eluted by incubating the beads with 100 units of AcTEV protease (Invitrogen) for 1.5 hours at 18ºC. After adding $CaCl_2$ to a final concentration of 2 mM, eluates were incubated with 200 µl of calmodulin-coated beads (Agilent) for 1 h at 4°C, with rotation. Beads were washed four times with 1 ml TMN150 buffer containing 2 mM $CaCl_2$, and then incubated with elution buffer (50 mM Tris pH 7.8, 100 mM NaCl, 5 mM EGTA, 0.1% NP40) for 10 min at 4°C. Eluted proteins were trichloroacetic acid (TCA) precipitated overnight at 4ºC and resolved on 4-12% NuPAGE BisTris gels (Invitrogen). Antibodies recognizing assembly factors were kindly provided by Katrin Karbstein. To generate the Rio1-TAP western blot results, a single blot was sequentially probed with antibodies.

HiSeq library preparation and sequencing

Reverse transcription reactions (20 µl final volume) were performed using Superscript III (Invitrogen), 30-70 fmol of purified ribosomal RNA and 2.5 µM of RT oligo (Supplementary Table S4). Samples were incubated at 45°C for 30 min. Subsequently, 10 U Exonuclease I and 25 U RNAseIf (New England Biolabs) was added to the reaction and incubated at 37°C for 30 min. The cDNAs were subsequently phenol/chloroform extracted, ethanol precipitated, resuspended in water and ligated to a 5' adapter sequence (see Supplementary Table S4) using CircLigase II (EpiCentre) according to the manufacturer's instructions. After PCR amplification, DNA products were

resolved on 2% MetaPhor agarose gels (Lonza) and 150-800 bp fragments were gel-purified using the MinElute kit (Qiagen) according to the manufacturer's instructions. Purified libraries were analyzed and quantified on a 2100 Bionalyzer (Agilent) using a High-Sensitivity DNA assay. Individual libraries were pooled appropriately based on concentration and barcoding, and paired-end sequencing was performed on a HiSeq 2000 system by Edinburgh Genomics (Edinburgh, UK) and BGI (Hong Kong).

Sequencing data analyses

Raw data processing was carried out using tools described in the pyCRAC software package version 1.1.9 (https://bitbucket.org/sgrann/pycrac (6)); the pySolexaBarcodeFilter tool was used to split reads based on barcoded indices. PyFastqDuplicateRemover was used to remove potential PCR duplicates using random nucleotide information in the 5' adapter sequence. Reads were mapped to the *Saccharomyces cerevisiae* 35S gene (RDN37-1) using Novoalign version 2.07 (www.novocraft.com). Only perfectly paired reads that mapped to a single position and completely overlapped with the 20S or 18S reference sequence were considered for further analyses. Read counts for the 18S and 20S-coding sequences, generated using pyPileup, were used to calculate RT drop-off rates.

The two-channel Poisson Expectation Maximization (TCP^{EM}) algorithm

The ChemModSeq protocol produces two channels of data, one series of read counts per nucleotide position on the rRNA for the chemically modified sample, and another for the control. In both cases, there are nucleotide positions where the polymerase is more likely to drop-off (high), and positions where drop-off is less likely (low). Assuming that high and low drop-off rates are approximately constant along the rRNA, we assigned each position to one of three categories: high drop-off in both channels (no assignment), low drop-off in both channels (unmodified), high drop-off in the modified channel and low in the control (modified). The drop-off rates (λ_1 λ_2) and the probabilities of a position belonging to a category (p_{i1} p_{i2} p_{i3} p_{i4}) were used to calculate the probability of the observed number of drop-offs (d_i) in modified and control

channels at position i given the observed read count (c_i) and (inferred) values of λ_1 λ_2 using a Poisson model. We calculate the likelihood as the product of these probabilities for all positions, and all categories. For example, for the first category where drop-off rates in both channels are λ_1 we compute:

$$p_{i1}\times\mathrm{Poisson}(d^{mod}_i;\ c^{mod}_i\lambda_1)\times\mathrm{Poisson}(d^{control}_i;\ c^{control}_i\lambda_1).$$

The assignment of positions to categories, and the calculation of λ_1 and λ_2 are performed iteratively using an expectation maximisation algorithm that maximises the likelihood of the data (7). In practice, the TCP^{EM} requires, on average, 22 iterations to converge (between 5 and 52), and on convergence the assignment of positions to categories is crisp: we use thresholds of 1.0 and 0.9 to decide class membership (from the range 0-1).

Sample preparation and quantitative label-free LC-MS

In solution digest was performed in a similar manner as described previously (8). Nano-UPLC-MS/MS analysis was performed using an on-line system consisting of a nano-pump Dionex Ultimate 3000 coupled to a QExactive Orbitrap instrument (Thermo-Fisher, UK) with a pre-column of 300 µm x 5 mm (Acclaim Pepmap, 5 µm particle size) connected to a column of 75 µm x 50 cm (Acclaim Pepmap, 3 µm particle size). Samples were analyzed on a 2 hours gradient in data dependent analysis (1 survey scan at 70k resolution followed by the top 5 MS/MS). Label-free quantitative analysis was performed using Progenesis (version 4.1 Nonlinear Dynamics, UK). Data from MS/MS spectra was searched using MASCOT Versions 2.4 (Matrix Science Ltd, UK) against the *Saccharomyces cerevisiae* subset of the NCBI protein database including contaminant for a total of 12 288 sequences) with maximum missed-cut value set to 2. Following features were used in all searches: i) variable methionine oxidation, ii) fixed cysteine carbamidomethylation, iii) precursor mass tolerance of 10 ppm, iv) MS/MS tolerance of 0.05 amu, v) significance threshold (p) below 0.05 (MudPIT scoring) and vi) final peptide score of 20 which correspond to less than 1%

FDR using decoy database search. The number of peptides used for quantification are listed in Supplementary Table S1.

SUPPLEMENTARY FIGURE LEGENDS

Figure S1. The C-terminal HTP tags generally do not affect protein function.

(A) Total RNA was extracted from the parental strain (BY4741) and HTP or TAP-tagged strain (indicated above the panel) and resolved on 1.25% agarose gels. To purify 80S-like complexes described by Strunk and colleagues (9), we grew *GAL-3HA::fap7* Fun12-TAP strain in glucose for four hours do deplete Fap7 and purified 20S associated with 80S-like complexes using Fun12 as a bait (lane 8). Mature rRNAs were detected by ethidium bromide (EtBr) staining. Pre-rRNA species were detected by northern blotting using oligonucleotides 003 and 004 (Supplementary Table S4). (B) Quantification of the 20S levels on the northern blot shown in (A). The 20S levels were normalized to 25S rRNA levels and then divided by the number obtained for the parental strain, BY4741. (C) Ten-fold serial dilutions of yeast strains grown on YPD. Yeast strains are indicated on the left of the panel. Note that Rio1-TAP grows very slow compared to the Rio1-HTP and parental strain.

Figure S2. The ChemModSeq protocol produces reproducible results.

Pearson correlation coefficients of ChemModSeq drop-off rate data comparing experimental replicates of DMS-modified and control samples.

Figure S3. Drop-off rates obtained from DMS, 1M7 and NAI experiments are uncorrelated with solvent accessibility of single stranded nucleotides.

Shown are average drop-off rates (n >=2) for nucleotides called modified by the TCP^{EM} algorithm in the 18S rRNA. Solvent accessibilities were calculated using the yeast 80S crystal structure data (10) using a probe size of 1.4$Å^2$. The R^2 value indicates the correlation coefficient between the solvent accessibility and drop-off rate.

Figure S4. Rio1-HTP transiently interacts with pre-40S complexes.

Extracts prepared from Rio1-HTP and Rio1-TAP strains were fractionated by

sucrose gradient (10%-50%) centrifugation. Twenty fractions were manually collected and analyzed by western blot using antibodies that recognize the Protein A moiety of the HTP and TAP tags (α-TAP), Rps14 or Nob1. The asterisk indicates a cross-reacting protein. RNA extracted from each fraction was resolved on 1.25% agarose gels. Mature rRNAs were detected by ethidium bromide (EtBr) staining. The 20S pre-rRNA was detected by northern blotting using oligonucleotide and 004 (Supplementary Table S4). Note that only a very small fraction of Rio1-HTP co-sedimented with 40S-sized particles, whereas Rio1-TAP almost exclusively co-sedimented with 40S complexes.

Figure S5. ChemModSeq accurately detects levels of natural modifications in pre-rRNAs.

(A) 20S pre-rRNAs associated with late pre-40S complexes have significantly higher levels of acp modification at U1191. Average U1191 drop-off rates were generated from at least two ChemModSeq experiments on unmodified 20S pre-rRNA. “Early” includes data from 20S pre-rRNAs isolated using Ltv1-HTP and Enp1-HTP as baits (n=2), “Middle” includes data from 20S pre-rRNAs isolated using Tsr1-HTP as bait (n=3) and “Late” includes data from 20S pre-rRNAs isolated using Fun12-TAP as bait (n=3). (B) As in (A) but showing data for the Dim1-dependent methylations at A1780 and A1781.

Figure S6. Subunit joining or tRNA binding to late pre-40S particles does not trigger restructuring of 3’ major domain.

(A). Secondary structure of the 18S 3’ major domain. Boxes and roman numbers highlight regions where most frequently differences in rRNA flexibility were found between early-middle and late particles. (B). The 3’ major domain of the 18S rRNA in empty and free 40S subunits is structurally more similar to 18S in translation initiation complexes. Middle pre-40S complexes, 80S translation initiation complexes and salt-washed, puromycin treated free 40S subunits were purified and incubated with 1M7 or solvent (DMSO, 5% final) for five minutes at room temperature. RNA was extracted and analyzed by primer extension using the oligonucleotides listed on the left side of each

panel (see Supplementary Table S4). Shown are the results for the regions in the 3' major domain where we could consistently detect differences in SHAPE reactivities between early-middle and late pre-40S complexes (indicated by brackets and roman numbers on the left side of each panel). Pre-40S and 80S translation initiation complexes were purified using Rio2 and Fun12 as baits, respectively. The positions of the modified nucleotides are indicated on the right side of each panel.

Figure S7. Primer extension analysis of 1M7 and NAI modified 20S and 18S rRNA.
(A-D) Primer extension results obtained for the 3' major domain for control (DMSO), 1M7 and NAI modified samples. Roman numbers highlight regions where most frequently differences in secondary structure were found between rRNA samples. (E) TCP^{EM} output of nucleotides most likely to be modified by NAI and/or 1M7 in the 3' major domain. Shown are the results for the Tsr1-associated 20S pre-rRNA. The blue (1M7), red (NAI) and purple (both) letters indicate nucleotides that the TCP^{EM} algorithm called modified by SHAPE reagents. Roman numbers highlight regions where most frequently differences in secondary structure were found between rRNA samples.

Figure S8. Nob1 does not play a (major) role in late head domain restructuring events. (A) Optimization of Nob1 depletion times. 3HA-Nob1 under the control of a *GAL* promoter is effectively depleted after four hours of growth in glucose. Western blots show the level of Fun12-TAP and 3HA-Nob1 in strains before (0) and after (2 and 4 hours) the shift to glucose. Tagged proteins were detected using an anti-HA antibody (Santa-Cruz) that recognized both ProtA and the 3xHA peptide. The four-hour time point was the chosen for the SHAPE experiments. (B) Pre-40S and 80S initiation complexes, purified from the parental and Nob1-depleted strains using Rio2-TAP or Fun12-TAP as baits, were modified with 1M7 *in vitro*. Extracted 20S and 18S rRNAs were analyzed by primer extension using oligonucleotide 69 (Supplementary Table S4). The analyses showed that 20S pre-rRNA purified from these Nob1-depleted complexes contained all the hallmarks of late pre-40S complexes: high levels of acp modification at U1191 and a reduction in

the flexibility of nucleotides in H37 and H35. This indicates that late restructuring steps can take place in the absence of Nob1.

Table S1. Number of peptides used for label free quantification

protein	Rio1-HTP	Rio2-HTP
Ltv1	1	3
Enp1	2	8
Pno1	1	1
Tsr1	4	14
Nob1	2	2
Rio1	3	N/A
Rio2	1	4

Table S3. *Saccharomyces cerevisiae* strains used in this study

Strain	Genotype	Reference
BY4741	MATa; *his3Δ1; leu2Δ0; met15Δ0; ura3Δ0*	(11)
D1092	as BY4741 but with *enp1*-HTP*::K.l.URA3*	(12)
YSG793	as BY4741 but with *nob1*-TAP*::K.l.URA3*	This study
D1077	as BY4741 but with *rio1*-HTP*::K.l.URA3*	(12)
YSG843	as BY4741 but with *rio1*-TAP*::K.l.URA3*	This study
D1079	as BY4741 but with *rio2*-HTP*::K.l.URA3*	(12)
YSG839	as BY4741 but with *rio2*-TAP*::K.l.URA3*	This study
D1081	as BY4741 but with *ltv1*-HTP::*K.l.URA3*	(12)
D1089	as BY4741 but with *tsr1-HTP::K.l.URA3*	(12)
YSG723	as BY4741 but with *fun12-TAP::K.l.HIS3*	This study
YSG773	as YSG723 but with *GAL::3HA-fap7::kanMX4*	This study
YSG808	as BY4741 but with *GAL::3HA-nob1::kanMX4 rio2-TAP::K.l.URA3*	This study
YSG779	as BY4741 but with *GAL::3HA-nob1::kanMX4 fun12-TAP ::K.l.HIS3*	This study
509	as BY4741 but with *YBR189w::kanMX4, YPL081w::HIS3* + *Yplac111-GAL-FLAG-RPS9A (LEU,CEN)*	P. Milkereit
603	as BY4741 but with *YHL015w::kanMX4* + *Yplac111-GAL-FLAG-RPS20 (LEU,CEN)*	P. Milkereit
693	as BY4741 but with *YNL178w::kanMX4* + *Yplac111-GAL-FLAG-RPS3 (LEU,CEN)*	P. Milkereit

Table S4 - Oligonucleotides used in this study

Oligonucleotides used for primer extension reactions.

Primer	Sequence (5'-3')
17	AATGCTCTATCCCCAGCACG
58	CGTCCTTGGCAAATGC
68	ATCGGTACTAGCGACGG
69	CCACTATTTAGTAGGTTAAGGTCTC
72	GGTTAGACTCGCTGGCT
80	CTAGTCGGCATAGTTTATGG
187	TACCACAGTTATACCATGTAGT
256	CAGCACAAGGCCATGCGATT
257	AAGCTCTCATGCTCTTGCCA
287	ACTTGCGCTTACTAGGAATTCC
288	AAGGGCATCACAGACCTGTT

Oligonucleotides used for northern blot analyses.

Primer	Sequence (5'-3')
003	TGTTACCTCTGGGCCC
004	CGGTTTTAATTGTCCTA

Oligonucleotides for HiSeq library preparation.

RT oligo	Sequence (5'-3')
PE_hexamer	CGTGTGCTCTTCCGATCTNNNNNN

5' adapter	Sequence (5'-3')
IDX1	5Phos/NCGTGATNNNNNNAGATCGGAAGAGCGTCGTGTAGGG/SpC3
IDX2	5Phos/NACATCGNNNNNNAGATCGGAAGAGCGTCGTGTAGGG/SpC3
IDX3	5Phos/NGCCTAANNNNNNAGATCGGAAGAGCGTCGTGTAGGG/SpC3
IDX4	5Phos/NTGGTCANNNNNNAGATCGGAAGAGCGTCGTGTAGGG/SpC3
IDX6	5Phos/NATTGGCNNNNNNAGATCGGAAGAGCGTCGTGTAGGG/SpC3

PCR primer	Sequence (5'-3')
P5	AATGATACGGCGACCACCGAGATCTACACTCTTTCCCTACACGACGCTCTTCCGATCT
BC1	CAAGCAGAAGACGGCATACGAGATCGTGATGTGACTGGAGTTCAGACGTGTGCTCTTCCGATCT
BC2	CAAGCAGAAGACGGCATACGAGATACATCGGTGACTGGAGTTCAGACGTGTGCTCTTCCGATCT
BC3	CAAGCAGAAGACGGCATACGAGATGCCTAAGTGACTGGAGTTCAGACGTGTGCTCTTCCGATCT
BC4	CAAGCAGAAGACGGCATACGAGATTGGTCAGTGACTGGAGTTCAGACGTGTGCTCTTCCGATCT
BC5	CAAGCAGAAGACGGCATACGAGATCACTGTGTGACTGGAGTTCAGACGTGTGCTCTTCCGATCT
BC6	CAAGCAGAAGACGGCATACGAGATATTGGCGTGACTGGAGTTCAGACGTGTGCTCTTCCGATCT

REFERENCES

1. Fatica, A., Oeffinger, M., Dlakic, M. and Tollervey, D. (2003) Nob1p is required for cleavage of the 3' end of 18S rRNA. *Mol. Cell. Biol.*, **23**, 1798–1807.

2. Sambrook, J. (1989) Molecular Clonig; a Laboratory Manual.

3. Beltrame, M., Henry, Y. and Tollervey, D. (1994) Mutational analysis of an essential binding site for the U3 snoRNA in the 5' external transcribed spacer of yeast pre-rRNA. *Nucleic Acids Res.*, **22**, 4057–4065.

4. Tollervey, D. (1987) A yeast small nuclear RNA is required for normal processing of pre-ribosomal RNA. *EMBO J*, **6**, 4169–4175.

5. Tollervey, D. and Mattaj, I.W. (1987) Fungal small nuclear ribonucleoproteins share properties with plant and vertebrate U-snRNPs. *EMBO J*, **6**, 469–476.

6. Webb, S., Hector, R.D., Kudla, G. and Granneman, S. (2014) PAR-CLIP data indicate that Nrd1-Nab3-dependent transcription termination regulates expression of hundreds of protein coding genes in yeast. *Genome Biol.*, **15**, R8.

7. Bishop, C.M. (2006) Pattern Recognition and Machine Learning Springer.

8. Le Bihan, T., Martin, S.F., Chirnside, E.S., van Ooijen, G., Barrios-Llerena, M.E., O'Neill, J.S., Shliaha, P.V., Kerr, L.E. and Millar, A.J. (2011) Shotgun proteomic analysis of the unicellular alga Ostreococcus tauri. *J Proteomics*, **74**, 2060–2070.

9. Strunk, B.S., Novak, M.N., Young, C.L. and Karbstein, K. (2012) A Translation-Like Cycle Is a Quality Control Checkpoint for Maturing 40S Ribosome Subunits. *Cell*, **150**, 111–121.

10. Ben-Shem, A., Garreau de Loubresse, N., Melnikov, S., Jenner, L., Yusupova, G. and Yusupov, M. (2011) The structure of the eukaryotic ribosome at 3.0 Å resolution. *Science*, **334**, 1524–1529.

11. Brachmann, C.B., Davies, A., Cost, G.J., Caputo, E., Li, J., Hieter, P. and Boeke, J.D. (1998) Designer deletion strains derived from Saccharomyces cerevisiae S288C: a useful set of strains and plasmids for PCR-mediated gene disruption and other applications. *Yeast*, **14**, 115–132.

12. Granneman, S., Petfalski, E., Swiatkowska, A. and Tollervey, D. (2010) Cracking pre-40S ribosomal subunit structure by systematic analyses of RNA-protein cross-linking. *EMBO J*, **29**, 2026–2036.

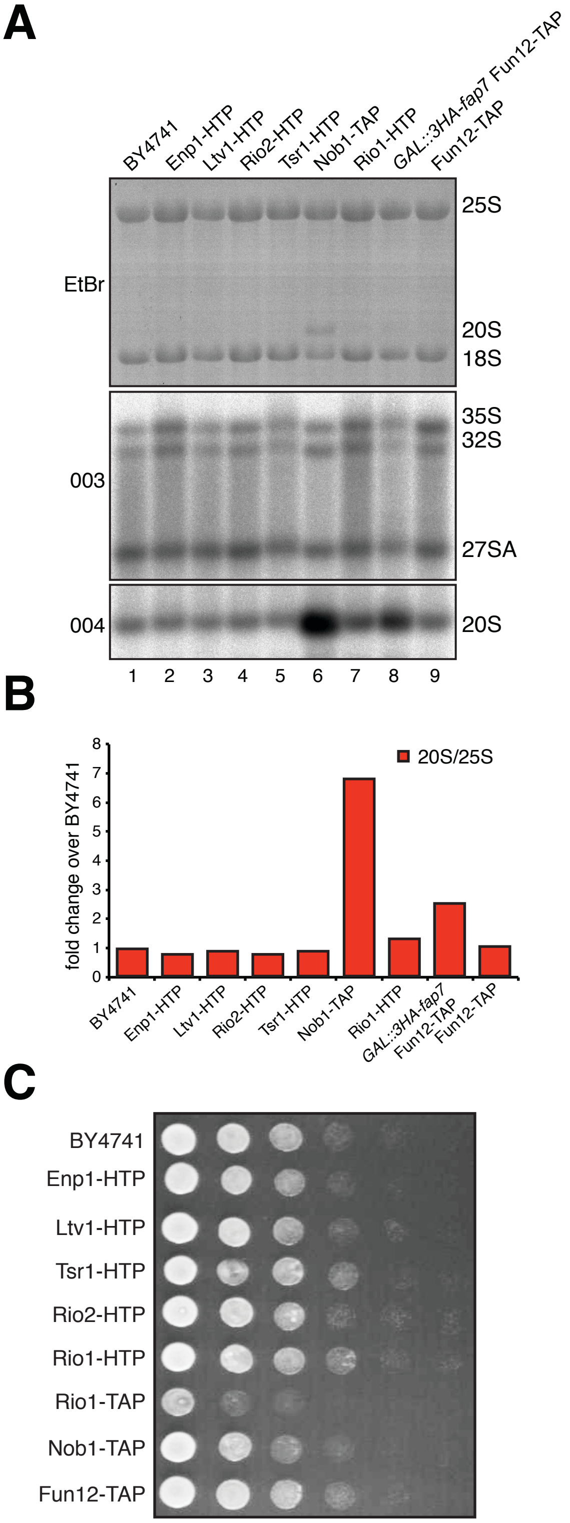

Hector et al Supplementary Figure S1

		DMS			Controls		
	replicate	1	2	3	1	2	3
DMS	1	1.0	0.84	0.90	0.69	0.51	0.67
	2	0.84	1.0	0.91	0.56	0.62	0.51
	3	0.90	0.91	1.0	0.54	0.48	0.55
Controls	1	0.69	0.56	0.54	1.0	0.63	0.87
	2	0.51	0.62	0.48	0.63	1.0	0.59
	3	0.67	0.51	0.54	0.87	0.59	1.0

Pearson correlation coefficients

Hector et al Supplementary Figure S2

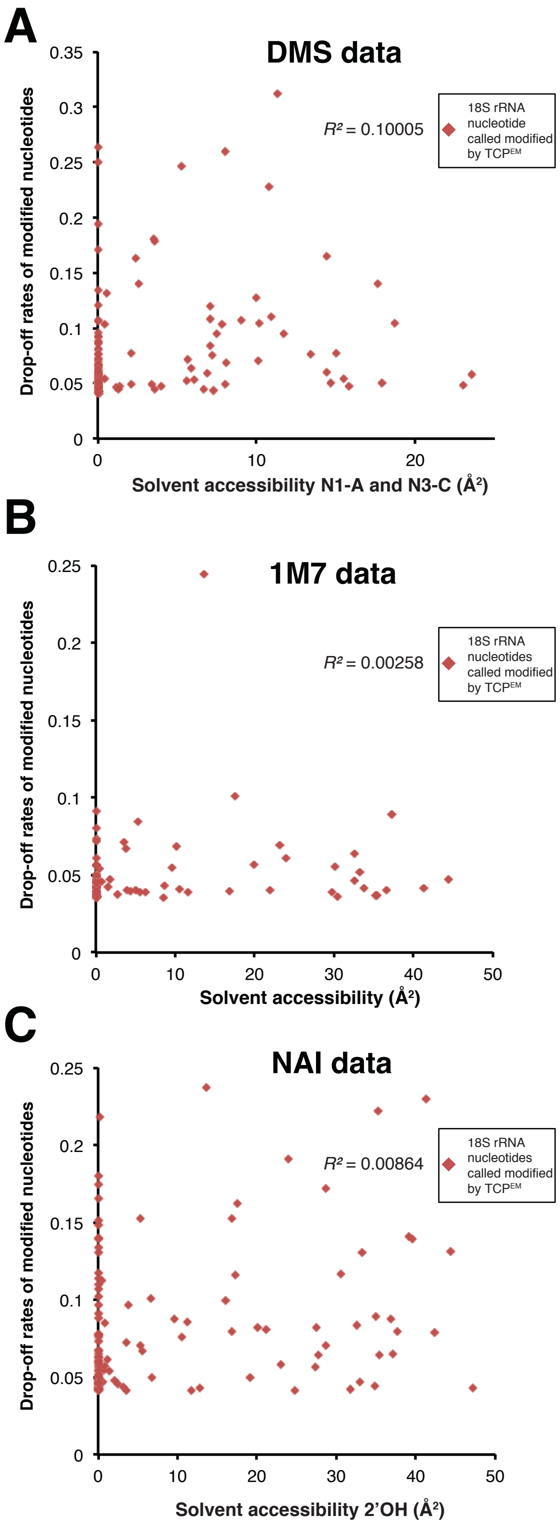

Hector et al Supplementary Figure S3

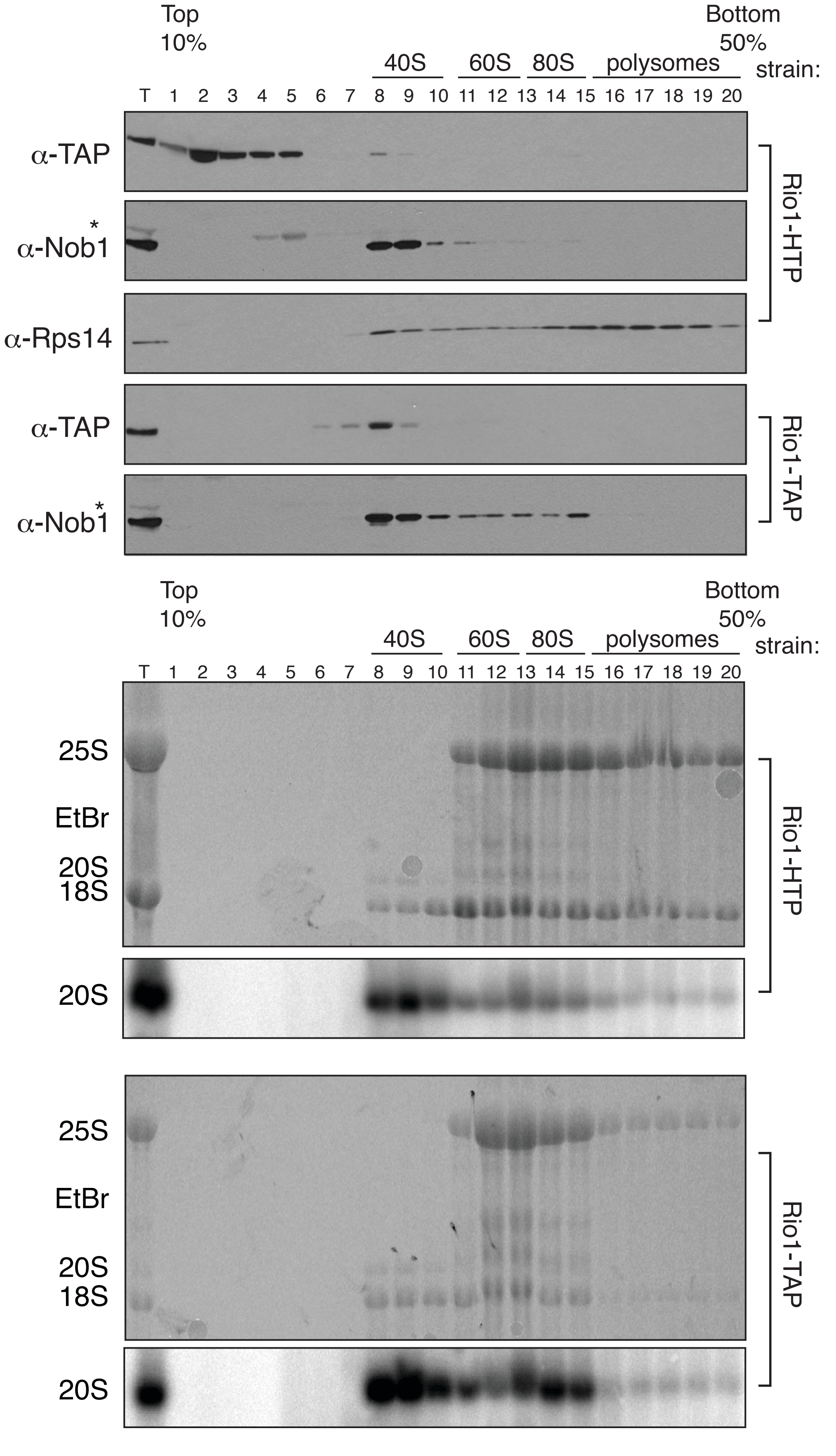

Hector et al Supplementary Figure S4

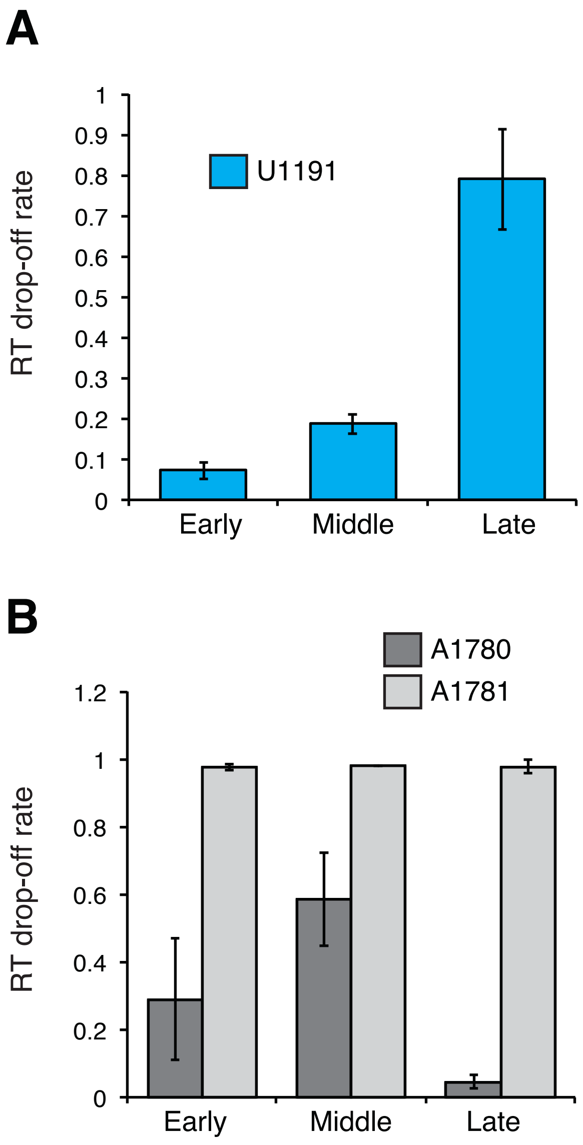

Hector et al Supplementary Figure S5

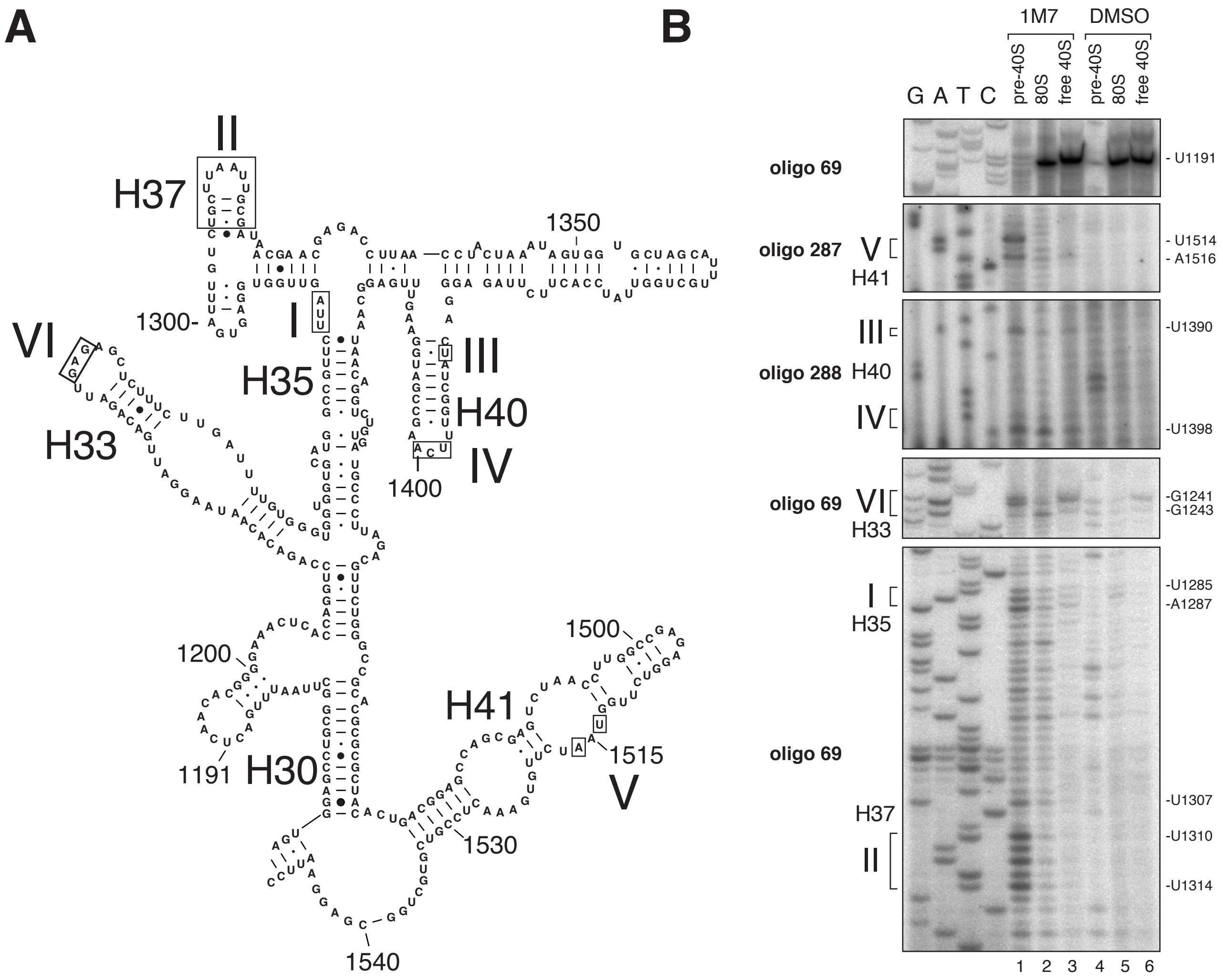

Hector et al Supplementary Figure S6

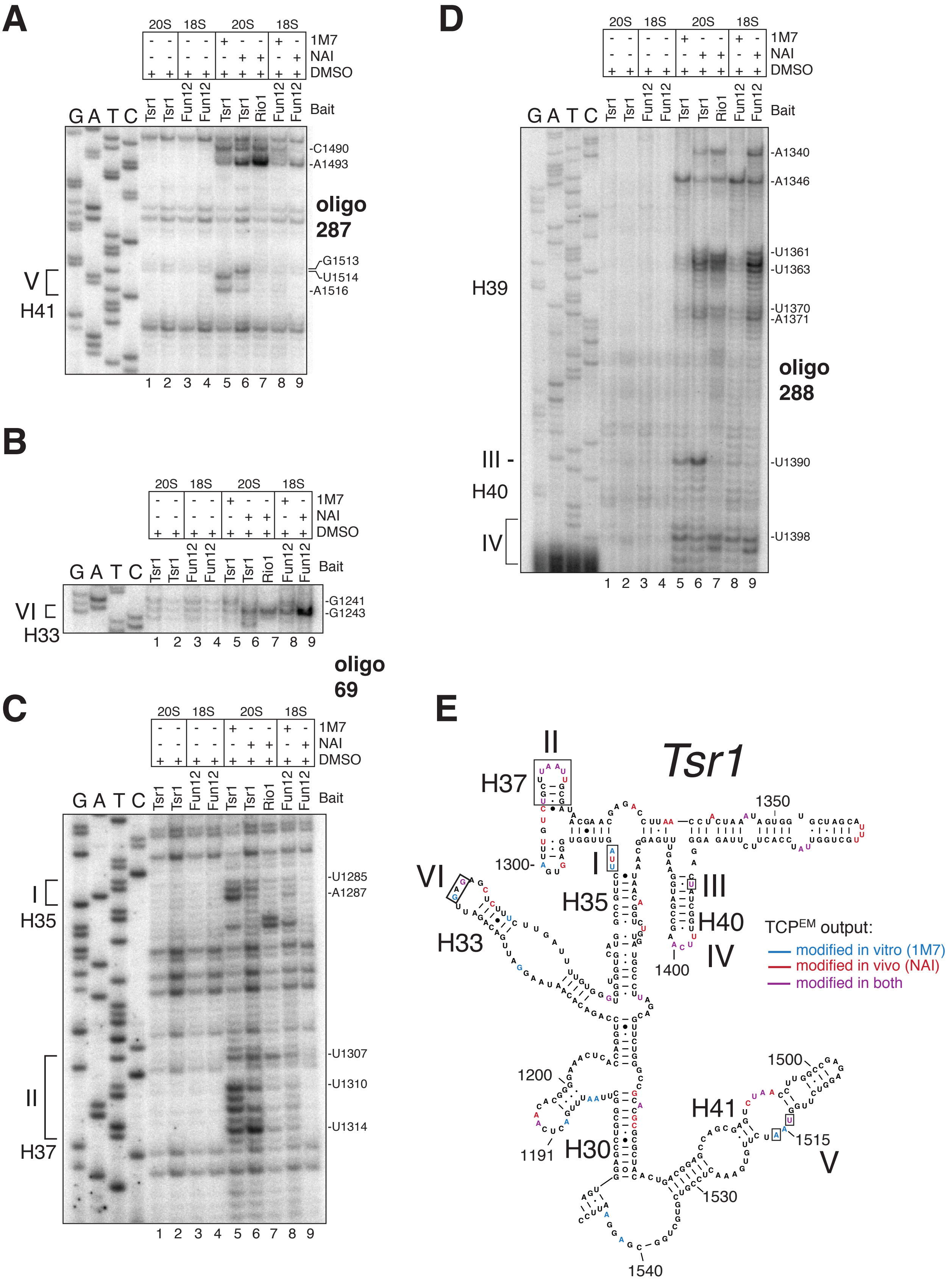

Hector et al Supplementary Figure S7

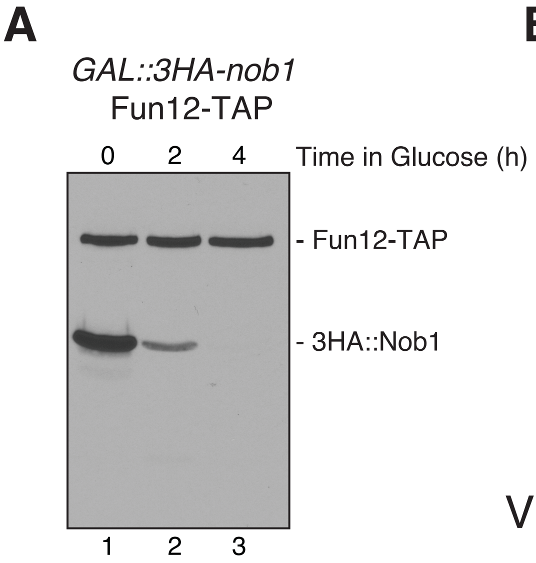

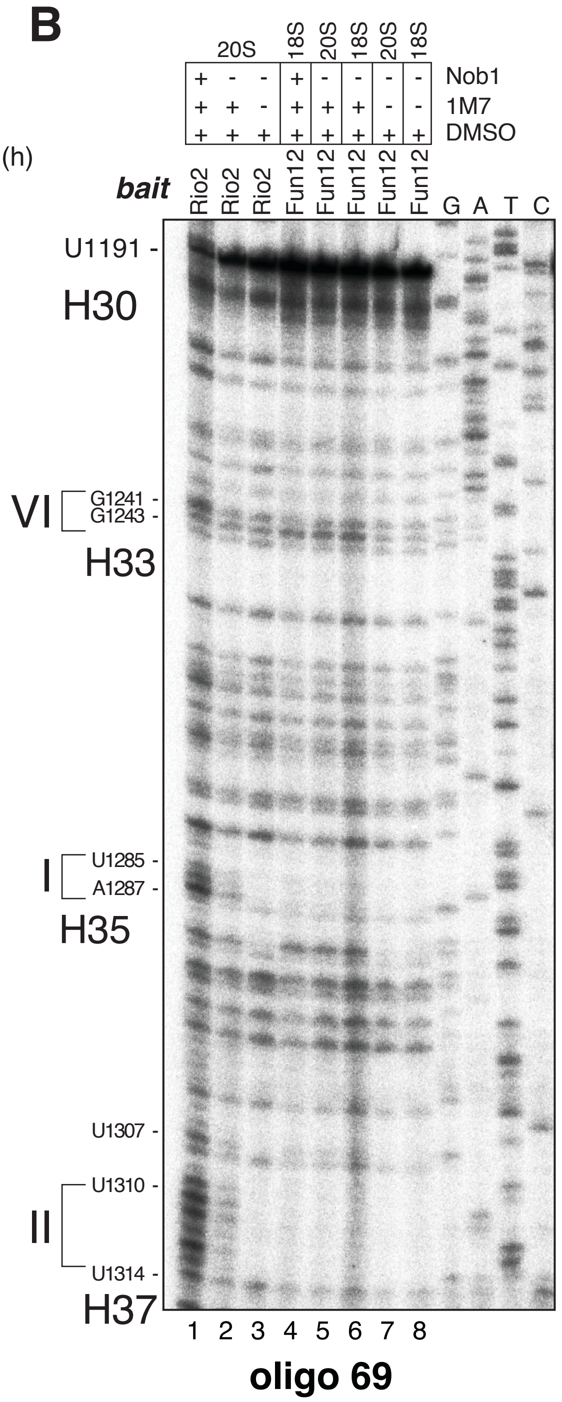

Hector et al Supplementary Figure S8
